# Supplementary material for: Differential regulation of mRNA fate by the human Ccr4-Not complex is driven by coding sequence composition and mRNA localization
Source: Genome Biol. 2021 Oct 6;22:284. doi: 10.1186/s13059-021-02494-w (PMC8496106; doi:10.1186/s13059-021-02494-w)
Supplement: Supplementary file 1 — Additional file 1. Supplementary figures. [file 13059_2021_2494_MOESM1_ESM.pdf]

Fig. S1

A.

Complex subunit	log2FC protein production
CNOT1	-3.10
CNOT2	-0.23
CNOT3	0.29
CNOT4	0.09
CNOT6/6L	ND
CNOT7	-0.82
CNOT8	ND
CNOT9	-1.64
CNOT10	-0.98
CNOT11	-0.11
TAB182	0.46

B.

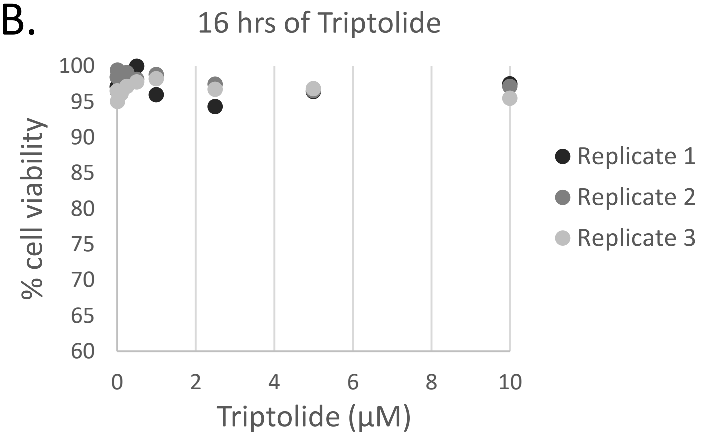

C.

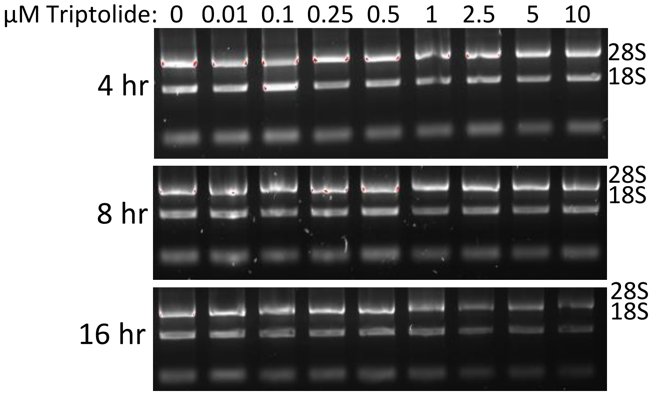

D.

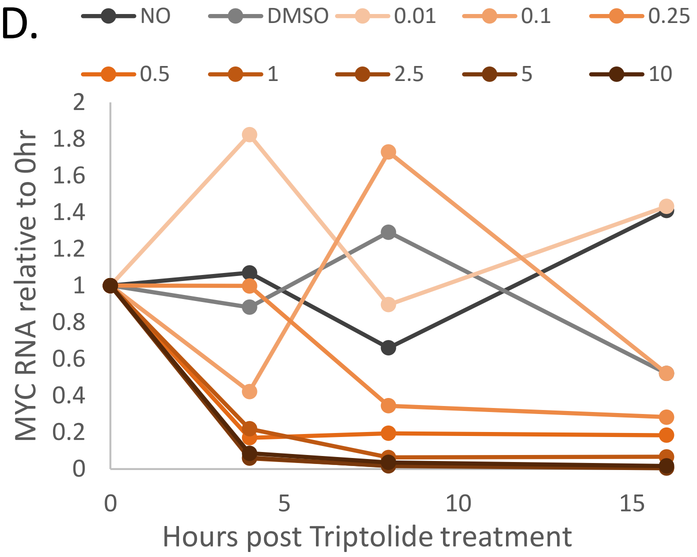

E.

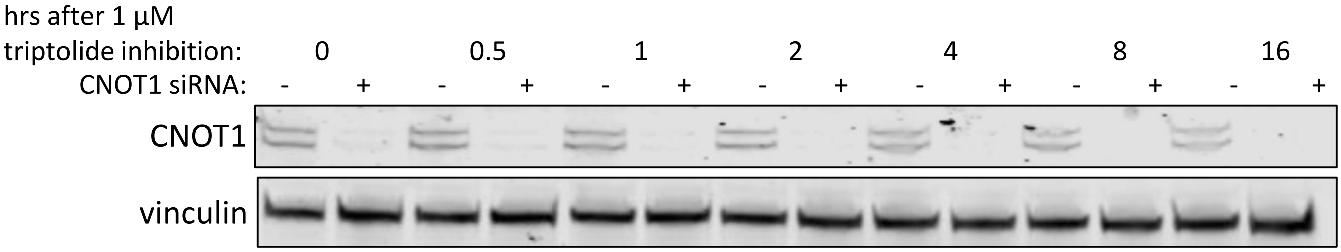

F.

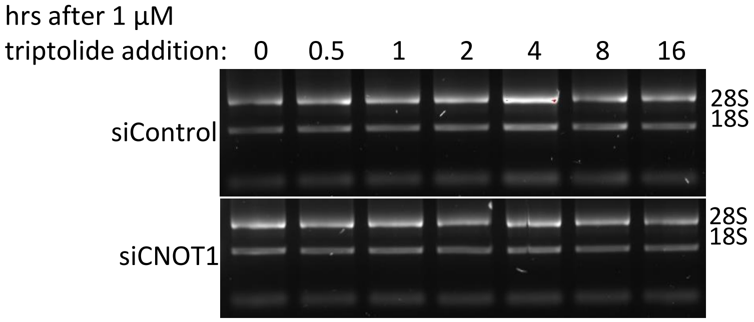

Fig. S1: Transcriptional inhibition experiment quality control. A. Change in the synthesis of Ccr4-Not complex subunits following CNOT1 depletion, data from the pulsed SILAC experiments detailed in Additional File 1: Fig. S8. ND = not detected. **B.** Triptolide does not negatively impact cell viability in the time period used. Cell viability was assessed using tryphan blue staining at a range of 0-10µM triptolide after 16 hrs treatment. **C.** RNA integrity from triptolide-treated cells was assessed by agarose gel. **D.** qPCR for MYC across the range of triptolide concentrations at 4, 8, and 16hrs post-treatment shown relative to the MYC level at 0 hrs. **E.** Representative western blot confirming CNOT1 knockdown is maintained throughout the triptolide treatment. **F.** Representative agarose gel showing RNA integrity across the timepoints and conditions.

Fig. S2

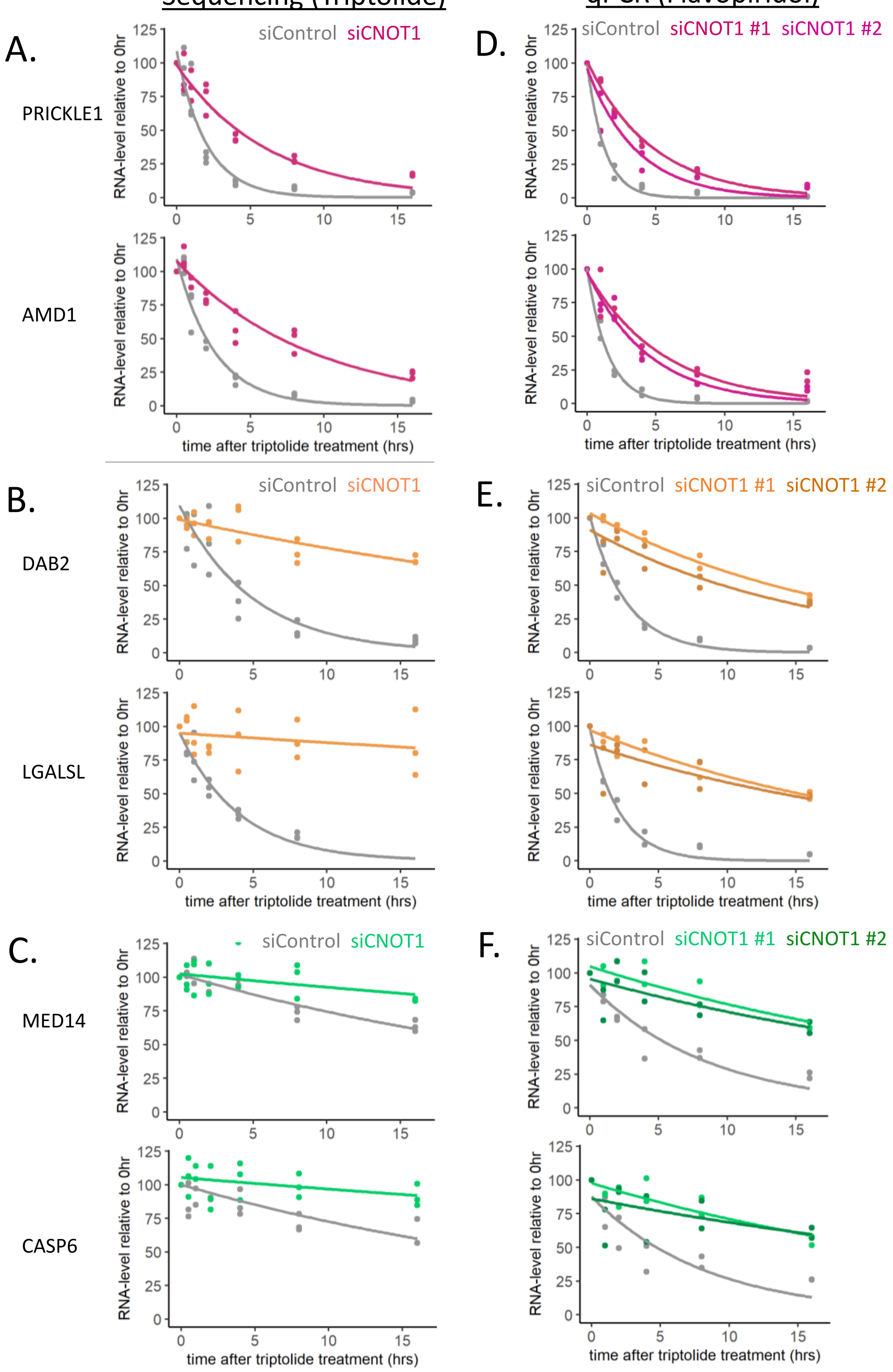

Fig. S2: Validation of mRNA half-lives. ABC. Example mRNA half-lives from the sequencing data used to determine global mRNA half-lives in the presence and absence of CNOT1. Examples are included for two mRNAs in each of the clusters in Fig. 1B. **DEF.** qPCR validations for the same mRNAs in ABC but using an alternative transcriptional inhibitor flavopiridol and an additional pool of siRNAs targeting CNOT1. This validates the mRNA half-lives for each of the clusters identified in Fig. 1B **D.** cluster 1. **E.** cluster 2 and **F.** cluster 3.

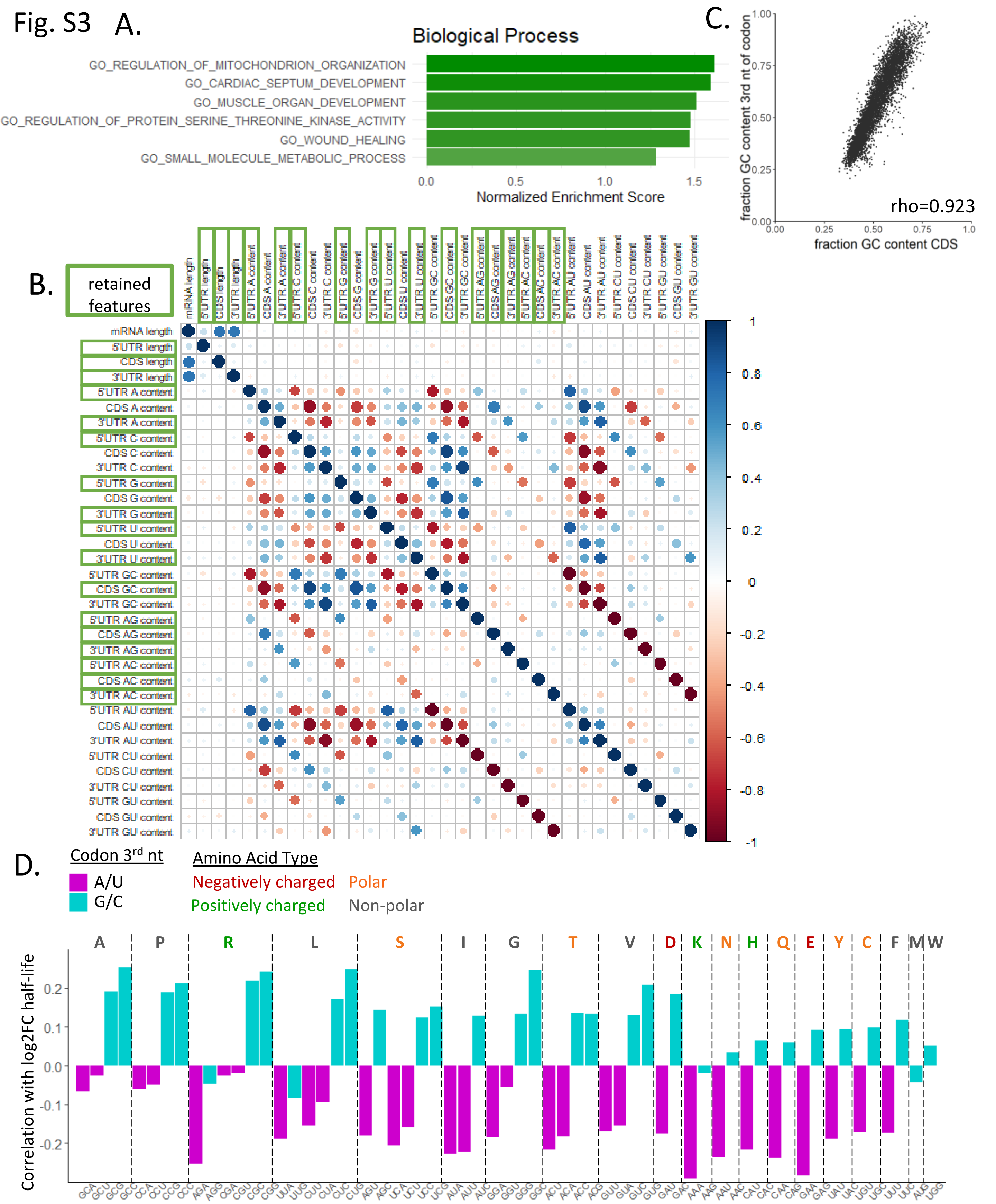

Fig. S3: mRNA feature analysis. A. The significant (adj.pval < 0.05) gene ontology biological process terms associated with an increase in mRNA half-life following CNOT1 depletion (conducted using the fgsea R package). **B.** Correlation matrix to identify highly correlated features. For features with a correlation coefficient > 0.75 only one of the features was retained. Retained features taken forward to feature importance analysis are highlighted. **C.** The correlation between the GC content of the CDS and the GC content of the 3rd nucleotide of the codons in the CDS. **D.** Correlation coefficient (Spearman's Rho) between the frequency of a given codon and the log2FC mRNA half-life (siCNOT1 / siControl) as in Fig. 1E, but reordered by the amino acid. Codons with an A/U at the 3rd nucleotide position are coloured in magenta and codons with a G/C at the 3rd nucleotide position are coloured in cyan. Amino acids are coloured by their charge/polarity type.

Fig. S4

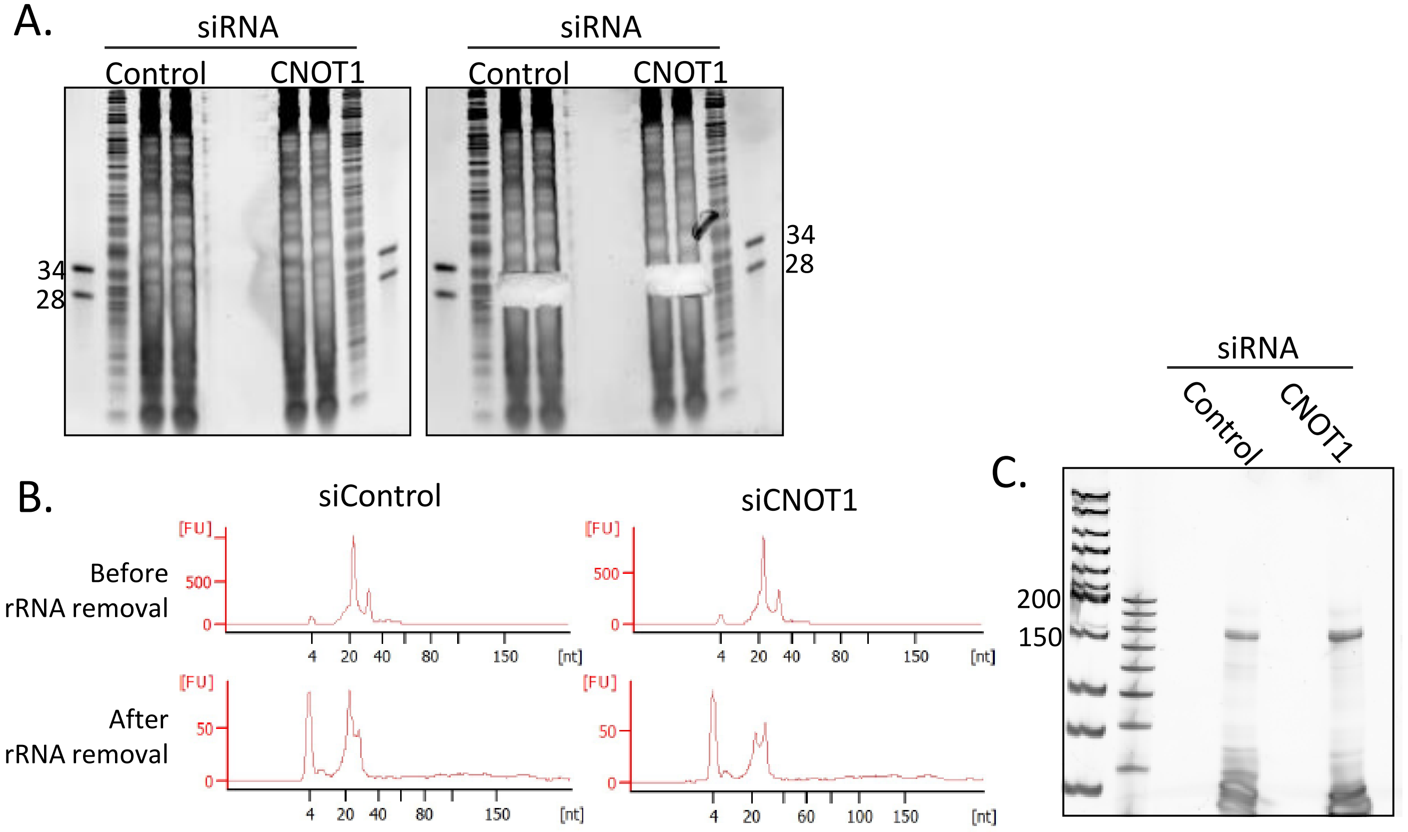

Fig. S4: Ribosome profiling experiment quality control. A-C. Representative examples of experimental quality control during RPF library production**. A.** TBE-Urea gel for extraction of RPFs (left=before, right= after extraction). Incision is made inclusive of the 28nt and exclusive of the 34nt marker position to avoid major contaminant rRNA fragments. **B.** Small RNA bioanalyzer chip traces of the extracted fragments before and after rRNA removal with Illumina RiboZero. **C.** TBE gel containing final small RNA libraries**.**

Fig. S5

A.

siRNA	Replicate	% rRNA
Control	1	4.98
Control	2	5.00
Control	3	4.12
CNOT1	1	11.05
CNOT1	2	6.43
CNOT1	3	8.40

B.

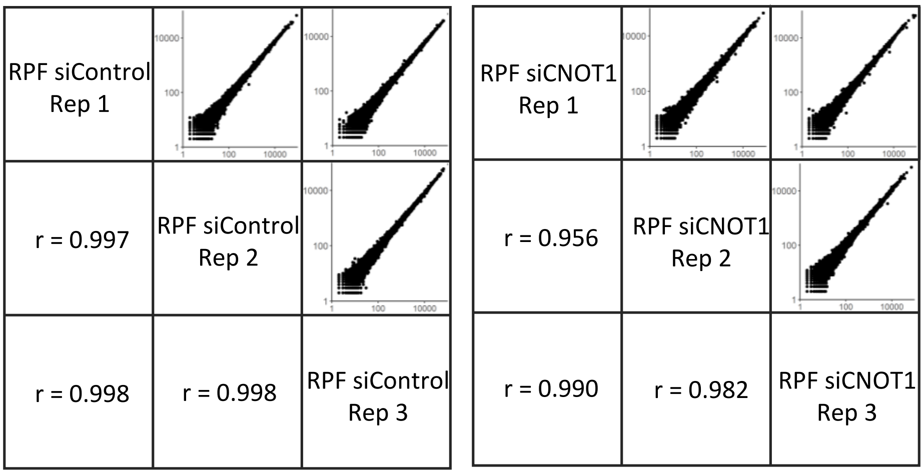

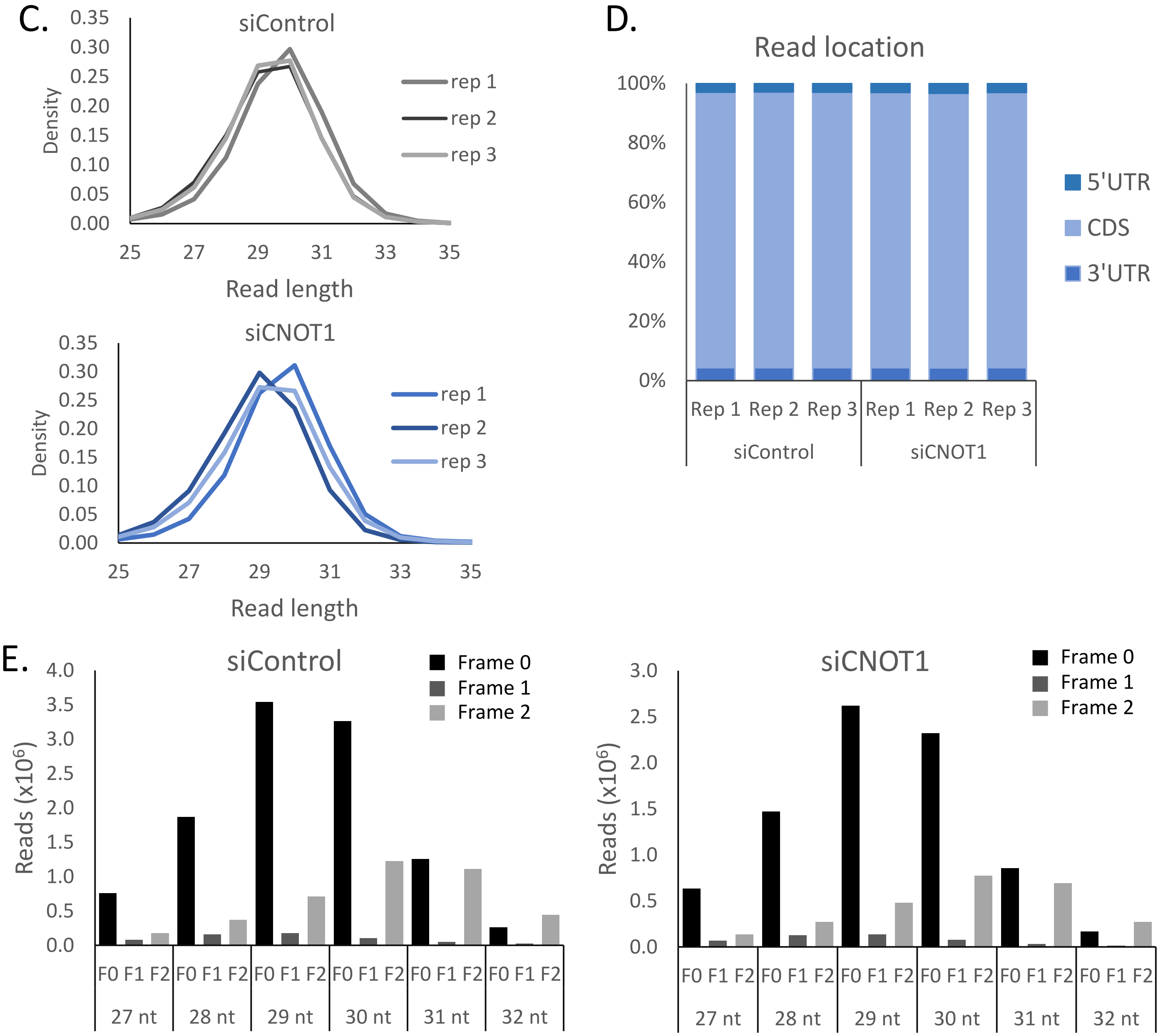

Fig. S5: Ribosome profiling data quality control. A. Proportion of reads that align to rRNA sequences in all samples. **B.** There is a high correlation across the biological replicates for the RPF sample read counts in both the control siRNA and CNOT1 siRNA treated samples. Pearson's r values are indicated. **C.** RPF samples show the expected read length distribution across the three replicates and both conditions. **D.** The majority of RPFs align to the CDS. Graph shows the proportion of RPFs aligning to the 5'UTR, CDS and 3'UTR. **E.** The frame distribution of read lengths 27 to 32 in control and CNOT1 siRNA treated samples. Data shown is for a representative replicate.

Fig. S6

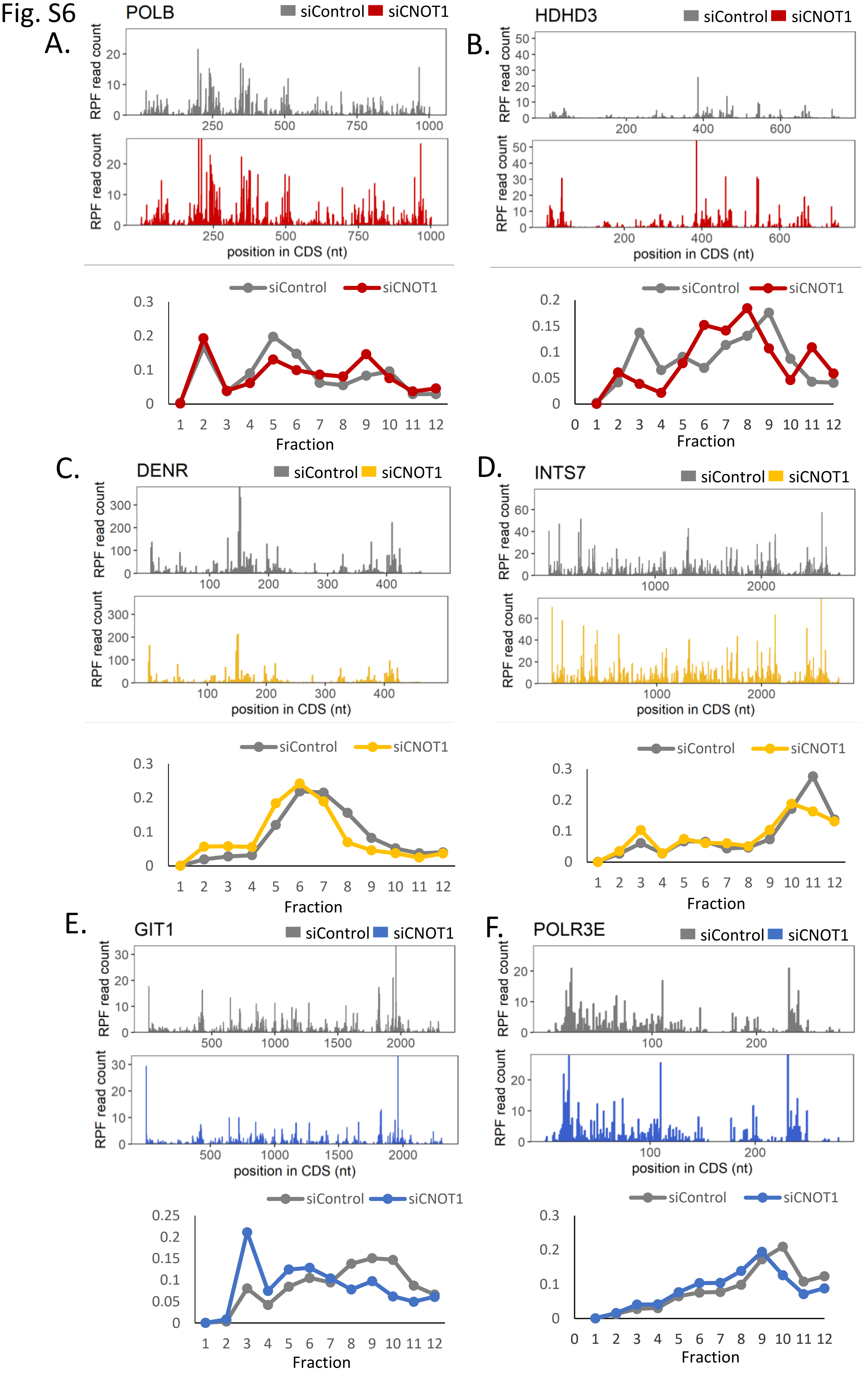
A.
POLB
siControl
siCNOT1
RPF read count
position in CDS (nt)
Fraction
B.
HDHD3
siControl
siCNOT1
RPF read count
position in CDS (nt)
Fraction
C.
DENR
siControl
siCNOT1
RPF read count
position in CDS (nt)
Fraction
D.
INTS7
siControl
siCNOT1
RPF read count
position in CDS (nt)
Fraction
E.
GIT1
siControl
siCNOT1
RPF read count
position in CDS (nt)
Fraction
F.
POLR3E
siControl
siCNOT1
RPF read count
position in CDS (nt)
Fraction

Fig. S6: Ribosome occupancy for individual mRNA examples. A-F. Average RPF read count from three biological replicates along the CDS of example mRNAs. This is shown for control (grey) and CNOT1 knockdown (coloured). Validation of ribosome profiling data (Fig. 2C) using qPCR along gradient fractions from an independent experiment with and without CNOT1 depletion (n=2, n=1 shown in Fig.2D-F). **AB.** POLB and HDHD3 that show increased TE after CNOT1 knockdown shift into polysomes. **CD.** DENR and INTS7 with no TE change after CNOT1 knockdown show minimal change in distribution across the gradient. **EF.** GIT1 and POLR3E that have decreased TE after CNOT1 knockdown shift towards the sub-polysomes.

Fig. S7

A.

Biological Process	Normalised Enrichment Score
NADH DEHYDROGENASE COMPLEX ASSEMBLY	1.94
MITOCHONDRIAL RESPIRATORY CHAIN COMPLEX ASSEMBLY	1.90
TRNA METABOLIC PROCESS	1.75
RESPIRATORY ELECTRON TRANSPORT CHAIN	1.68
REGULATION OF CENTROSOME DUPLICATION	1.67
TRNA MODIFICATION	1.67
REGULATION OF SISTER CHROMATID SEGREGATION	1.66
CENTROSOME DUPLICATION	1.65
MITOCHONDRIAL GENE EXPRESSION	1.63
TRNA PROCESSING	1.62
CARDIAC CONDUCTION	-2.00
VASCULAR ENDOTHELIAL GROWTH FACTOR RECEPTOR SIGNALING PATHWAY	-2.01
NEURON PROJECTION GUIDANCE	-2.03
SECOND MESSENGER MEDIATED SIGNALING	-2.03
REGULATION OF RESPONSE TO WOUNDING	-2.04
REGULATION OF SYNAPSE STRUCTURE OR ACTIVITY	-2.05
SYNAPSE ORGANIZATION	-2.05
CYCLIC NUCLEOTIDE MEDIATED SIGNALING	-2.07
CELL CELL ADHESION VIA PLASMA MEMBRANE ADHESION MOLECULES	-2.11
EXTRACELLULAR STRUCTURE ORGANIZATION	-2.22

B.

Molecular Function	Normalised Enrichment Score
TRNA BINDING	1.77
DNA BINDING TRANSCRIPTION REPRESSOR ACTIVITY	1.63
PASSIVE TRANSMEMBRANE TRANSPORTER ACTIVITY	-2.00
ACTIVE ION TRANSMEMBRANE TRANSPORTER ACTIVITY	-2.00
GATED CHANNEL ACTIVITY	-2.03
PROTEIN KINASE ACTIVITY	-2.03
STRUCTURAL MOLECULE ACTIVITY	-2.03
MOLECULAR TRANSDUCER ACTIVITY	-2.11
PROTEIN TYROSINE KINASE ACTIVITY	-2.12
TRANSMEMBRANE RECEPTOR PROTEIN KINASE ACTIVITY	-2.15
GROWTH FACTOR BINDING	-2.24
EXTRACELLULAR MATRIX STRUCTURAL CONSTITUENT	-2.77

C.

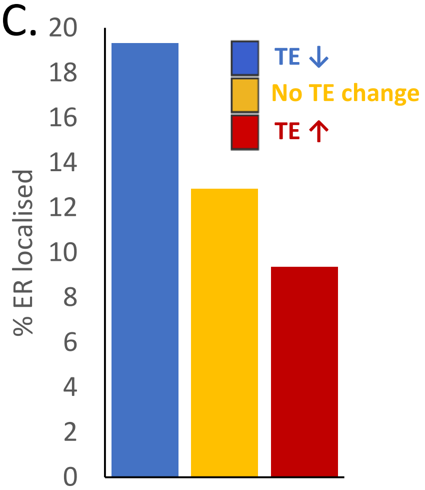

D.

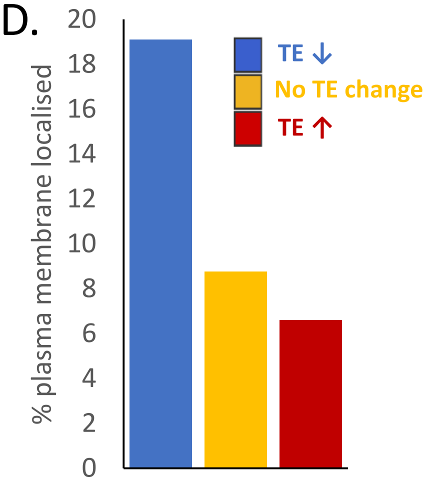

Fig. S7: Gene set enrichment analysis. AB. All mRNAs were ordered by the log2FC TE (siCNOT1/siControl) and gene set enrichment analysis conducted on the ranked list using the fgsea R package for **A.** biological processes & **B.** Molecular function. Red highlights terms with an enrichment associated with increased TE and blue highlights terms linked with a decreased TE following CNOT1 depletion. Up to a maximum of 10 significant terms (adj.pval < 0.05) are shown in this figure, the complete list of results are in Supplemental Table 3. **CD.** LOPIT data (from U2OS cells, (88)) was used to examine where the encoded proteins localise. mRNAs with decreased TE following CNOT1 depletion are enriched for proteins that localise to **C.** the ER & **D.** the plasma membrane.

Fig. S8

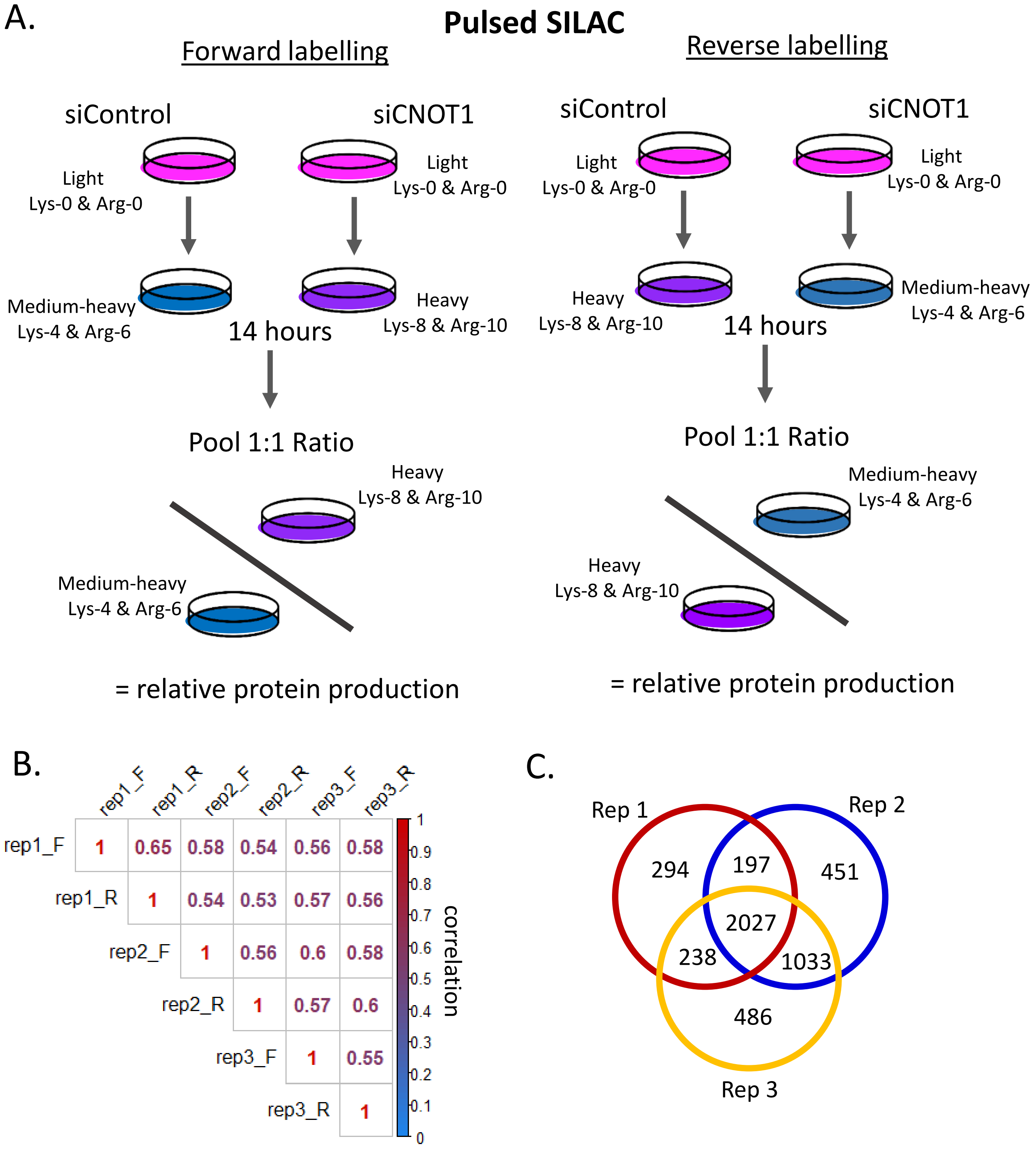

Fig. S8: Pulsed SILAC quality control. A. For each biological replicate of pulsed SILAC, two technical replicates are required – forward and reverse labelling. Light, medium-heavy and heavy indicate the isotope of lysine and arginine present in the medium. Cells were treated with control siRNA or CNOT1-targeting siRNA for 30 hrs, before addition of medium-heavy/heavy media to cells for 14hrs. **B.** Correlations between forward and reverse labelling methods and biological replicates for pulsed SILAC data. Shown is the Pearson correlation coefficient. **C.** Venn diagram of the overlap of proteins detected in each replicate. For each replicate only proteins detected in both the forward and reverse labelling were used.

Fig. S9

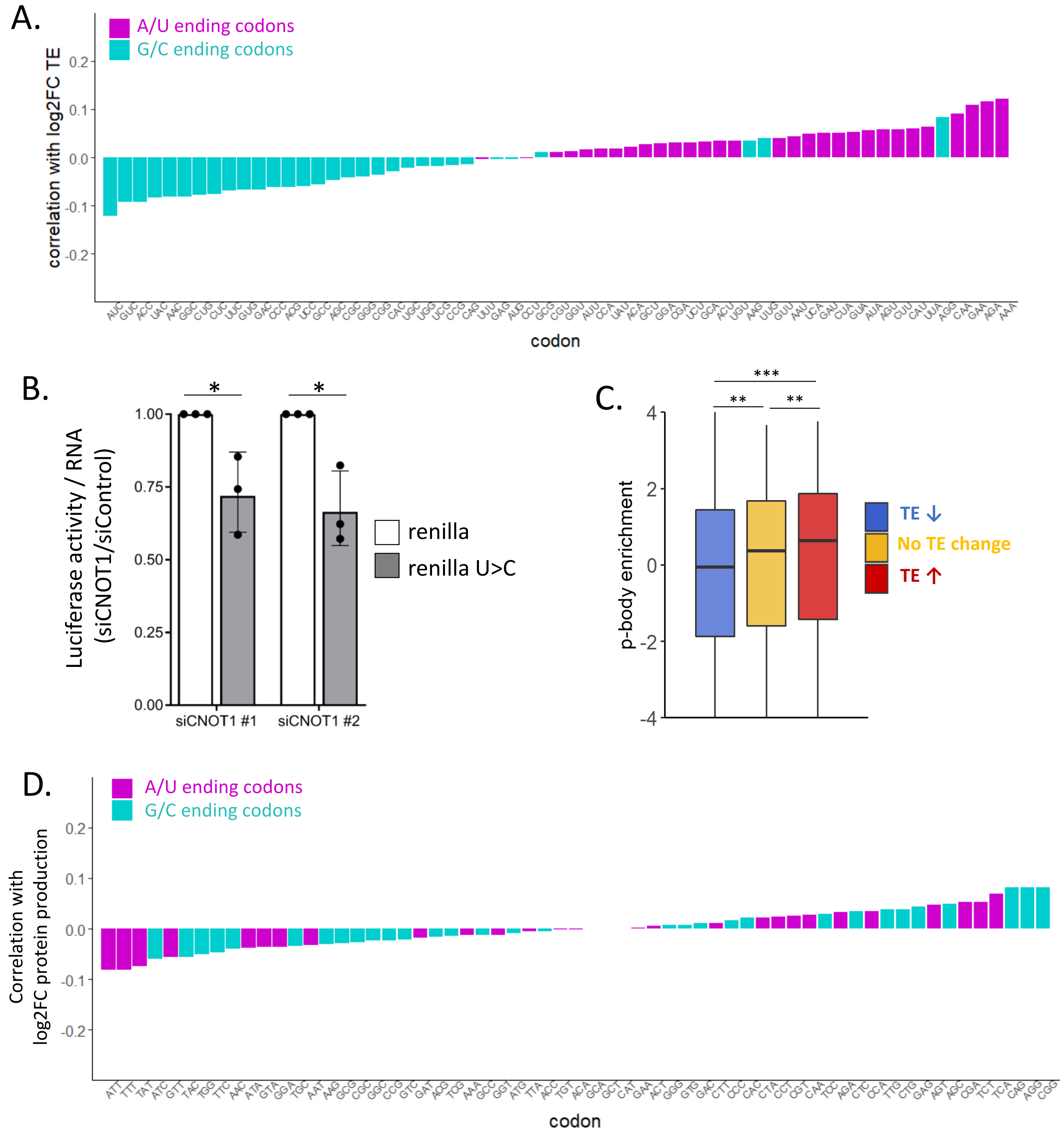

Fig. S9 Codon frequency correlation with log2TE. A. Correlation coefficient (Spearman's Rho) between the frequency of a given codon in an mRNA and the change in translational efficiency (siCNOT1/siControl) as determined by the ribosome profiling experiments. Codons with an A/U at the 3rd nucleotide position are coloured in magenta and codons with a G/C at the 3rd nucleotide position are coloured in cyan. **B.** The codons identified as most negatively correlated with the log2FC TE in (A) – AUC/GUC/ACC were introduced at synonymous positions in the Renilla CDS (AUU/GUU/ACU). Renilla translational efficiency is calculated by the luciferase activity / luciferase RNA level (after normalisation to Firefly luciferase transfection control). The graph shows the translational efficiency change with CNOT1 depletion and the difference between the original Renilla CDS and the codon altered CDS. **C.** mRNAs with increased TE when CNOT1 is depleted are enriched in p-bodies in control conditions (data from HEK293 cells, (94)). **D.** Correlation coefficient (Spearman's Rho) between the frequency of a given codon in an mRNA and the change in protein production (siCNOT1/siControl) as determined by pulsed SILAC experiments. Codons with an A/U at the 3rd nucleotide position are coloured in magenta and codons with a G/C at the 3rd nucleotide position are coloured in cyan.

Fig. S10

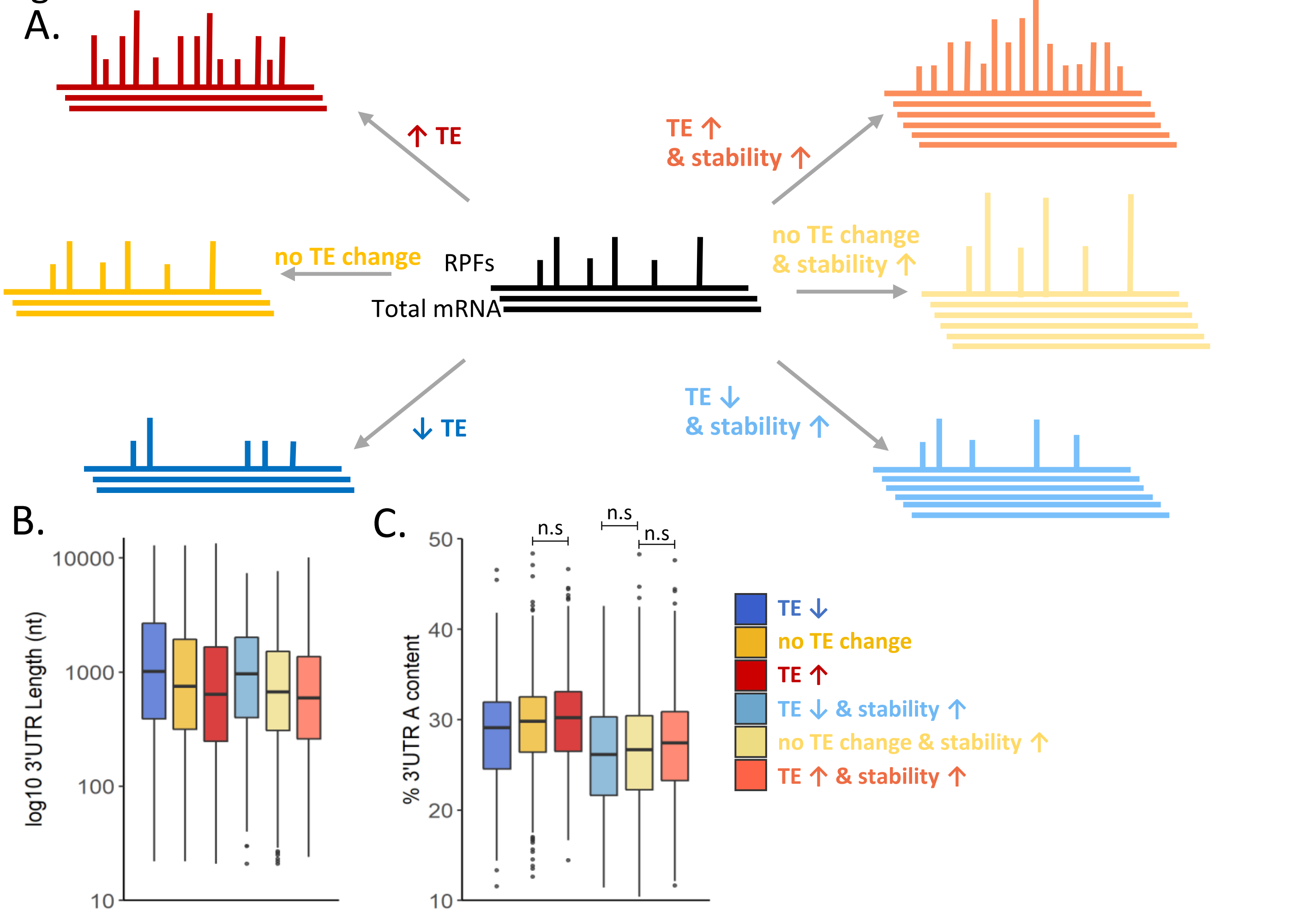

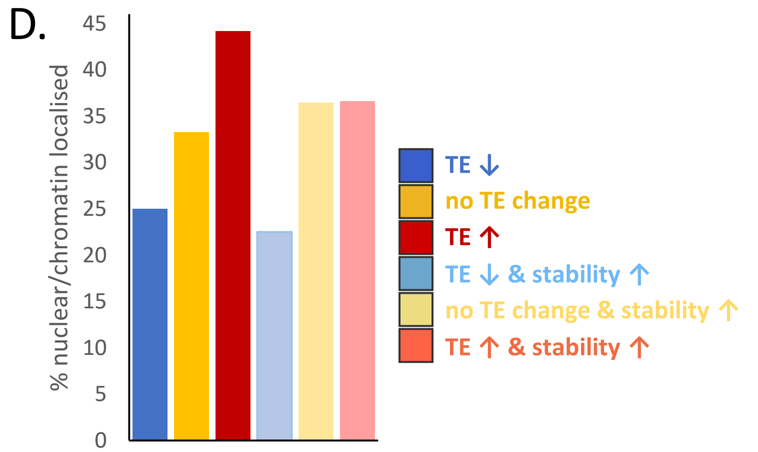

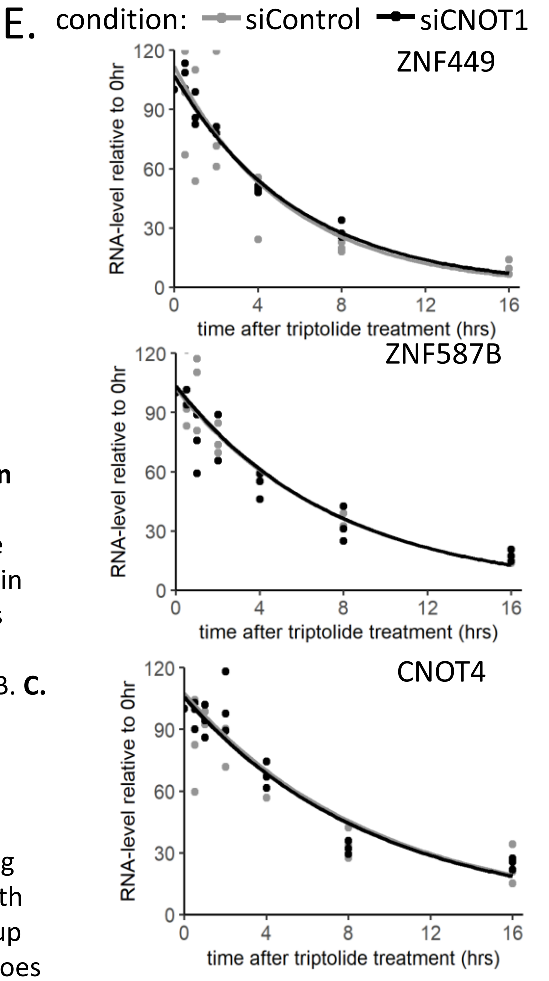

Fig. S10: mRNAs only upregulated translationally after CNOT1 depletion encode nuclear proteins. A. Diagram to illustrate the categorisation of mRNAs in Fig. 4A by how the Ccr4-Not complex differentially impacts the translation and/or stability of the mRNA **B.** Distribution of 3'UTR lengths in the groups of mRNAs differentially regulated by the Ccr4-Not complex as classified in Fig. 4A. All comparisons were determined to be statistically significant using the dunn test and are shown in Additional File 9: Fig. S2B. **C.** The 3'UTR A content for the distinct groups of mRNAs. Statistical comparisons for A & B are shown in Additional File 9: Fig. S2BC. Only the non-significant comparisons are displayed for clarity and all statistics are presented in Additional File 2: Fig. S2B. **D.** Percentage of the groups of mRNAs in Fig.4A that localise to the nucleus/chromatin, determined using LOPIT data (from U2OS cells, (88)). **E.** Example mRNA stability profiles with control or CNOT1-targeting siRNA treatment for mRNAs in the TE ↑ group (dark red, Fig. 4A). They have a short half-life in control conditions that does not change with CNOT1 knockdown.

Fig. S11

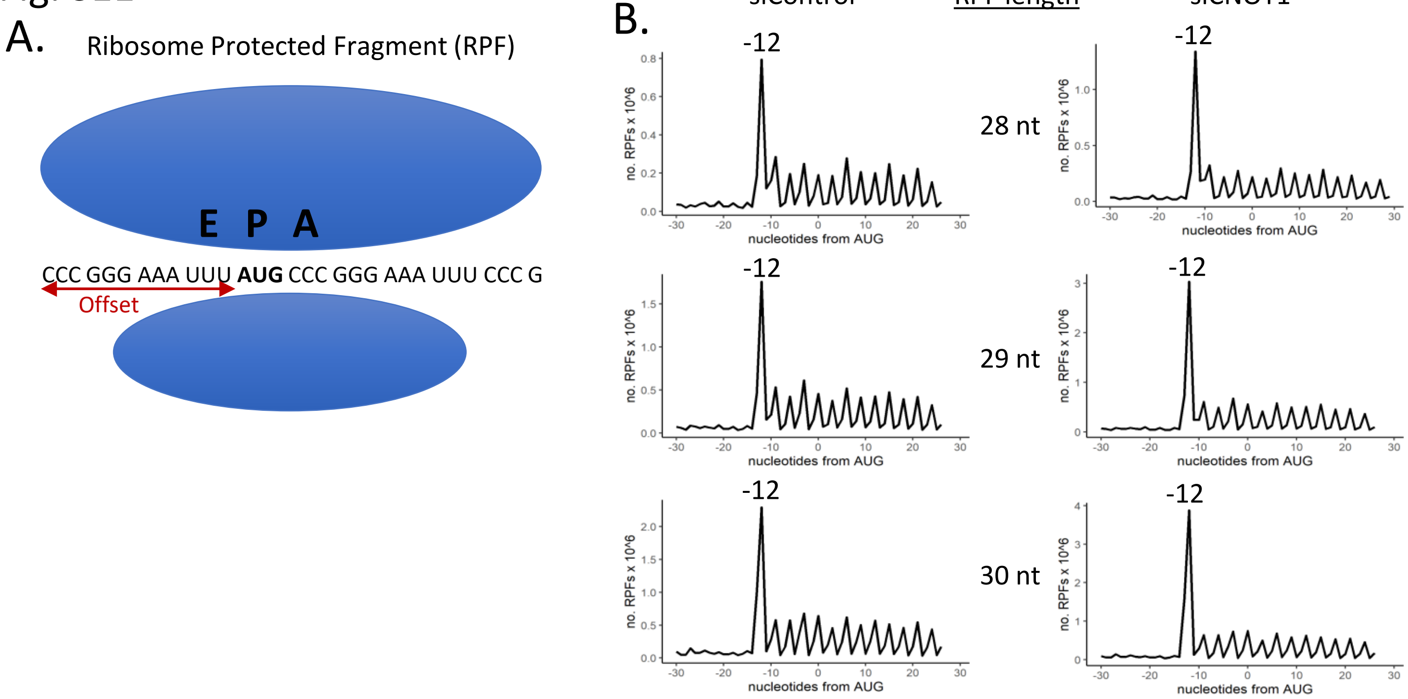

Fig. S11: Precise ribosome residency. A. To be able to assign the location of the E/P/A tRNA binding sites of the ribosome, an offset needs to be applied from the start of the read. The start codons have high ribosome occupancy so by looking at the positions of the read starts around this region, as in B, the offset to position the peak at the P-site. **B.** Plots show the RPF read distribution around the AUG start codon of all transcripts for a representative replicate. P-site offset is determined to be 12nt and thus the E-site offset is 9nt and A-site offset 15nt.
