# Supplementary material for: Differential regulation of mRNA fate by the human Ccr4-Not complex is driven by coding sequence composition and mRNA localization
Source: Genome Biol. 2021 Oct 6;22:284. doi: 10.1186/s13059-021-02494-w (PMC8496106; doi:10.1186/s13059-021-02494-w)
Supplement: Supplementary file 9 — Additional file 9. Statistics for Main Figures 4 and 5C and Additional file 1: S10 B/C. [file 13059_2021_2494_MOESM9_ESM.pdf]

A. Protein production (Fig. 4B)

No TE change	TE ↓	TE ↑ & stability ↑	No TE change & stability ↑	TE ↓ & stability ↑	
*	***	**	*	n.s	TE ↑
	**	***	***	*	No TE change
		***	***	***	TE ↓
			*	**	TE ↑ & stability ↑
				*	No TE change & stability ↑

*** p.adj < 0.001
** p.adj < 0.01
* p.adj < 0.1

B. CDS length (Fig. 4D)

No TE change	TE ↓	TE ↑ & stability ↑	No TE change & stability ↑	TE ↓ & stability ↑	
*	***	***	***	*	TE ↑
	***	***	***	***	No TE change
		***	***	***	TE ↓
			**	***	TE ↑ & stability ↑
				**	No TE change & stability ↑

*** p.adj < 0.001
** p.adj < 0.01
* p.adj < 0.1

C. GC content (Fig. 4E)

No TE change	TE ↓	TE ↑ & stability ↑	No TE change & stability ↑	TE ↓ & stability ↑	
n.s	**	***	***	***	TE ↑
	**	***	***	***	No TE change
		***	***	***	TE ↓
			n.s	n.s	TE ↑ & stability ↑
				**	No TE change & stability ↑

*** p.adj < 0.001
** p.adj < 0.01
* p.adj < 0.1

D. AG content (Fig. 4F)

No TE change	TE ↓	TE ↑ & stability ↑	No TE change & stability ↑	TE ↓ & stability ↑	
**	***	***	***	***	TE ↑
	***	***	***	***	No TE change
		n.s	*	***	TE ↓
			n.s	***	TE ↑ & stability ↑
				***	No TE change & stability ↑

*** p.adj < 0.001
** p.adj < 0.01
* p.adj < 0.1

Additional File 2, Fig. S1: Statistics for Main Figure 4. Dunn test was used with Benjamini-Hochberg correction for multiple hypothesis testing correction.

A. Disorder promoting AAs (Fig. 5C)

No TE change	TE ↓	TE ↑ & stability ↑	No TE change & stability ↑	TE ↓ & stability ↑	
***	***	***	***	***	TE ↑
	***	***	***	***	No TE change
		n.s	n.s	n.s	TE ↓
			n.s	n.s	TE ↑ & stability ↑
				n.s	No TE change & stability ↑

*** p.adj < 0.001
** p.adj < 0.01
* p.adj < 0.1

B. 3'UTR length (Fig. S10B)

No TE change	TE ↓	TE ↑ & stability ↑	No TE change & stability ↑	TE ↓ & stability ↑	
*	***	***	***	*	TE ↑
	***	***	***	***	No TE change
		***	***	***	TE ↓
			**	***	TE ↑ & stability ↑
				**	No TE change & stability ↑

*** p.adj < 0.001
** p.adj < 0.01
* p.adj < 0.1

C. 3'UTR A content (Fig. S10C)

No TE change	TE ↓	TE ↑ & stability ↑	No TE change & stability ↑	TE ↓ & stability ↑	
n.s	**	***	***	***	TE ↑
	*	***	***	***	No TE change
		*	***	***	TE ↓
			n.s	*	TE ↑ & stability ↑
				n.s	No TE change & stability ↑

*** p.adj < 0.001
** p.adj < 0.01
* p.adj < 0.1

Additional File 2, Fig. S2: Statistics for Main Figure 5C Additional File 1 S10BC.
Dunn test was used with Benjamini-Hochberg correction for multiple hypothesis testing correction.
