# Supplementary material for: Differential regulation of mRNA fate by the human Ccr4-Not complex is driven by coding sequence composition and mRNA localization
Source: Genome Biol. 2021 Oct 6;22:284. doi: 10.1186/s13059-021-02494-w (PMC8496106; doi:10.1186/s13059-021-02494-w)
Supplement: Supplementary file 10 — Additional file 10. Uncropped Western blots. [file 13059_2021_2494_MOESM10_ESM.pdf]

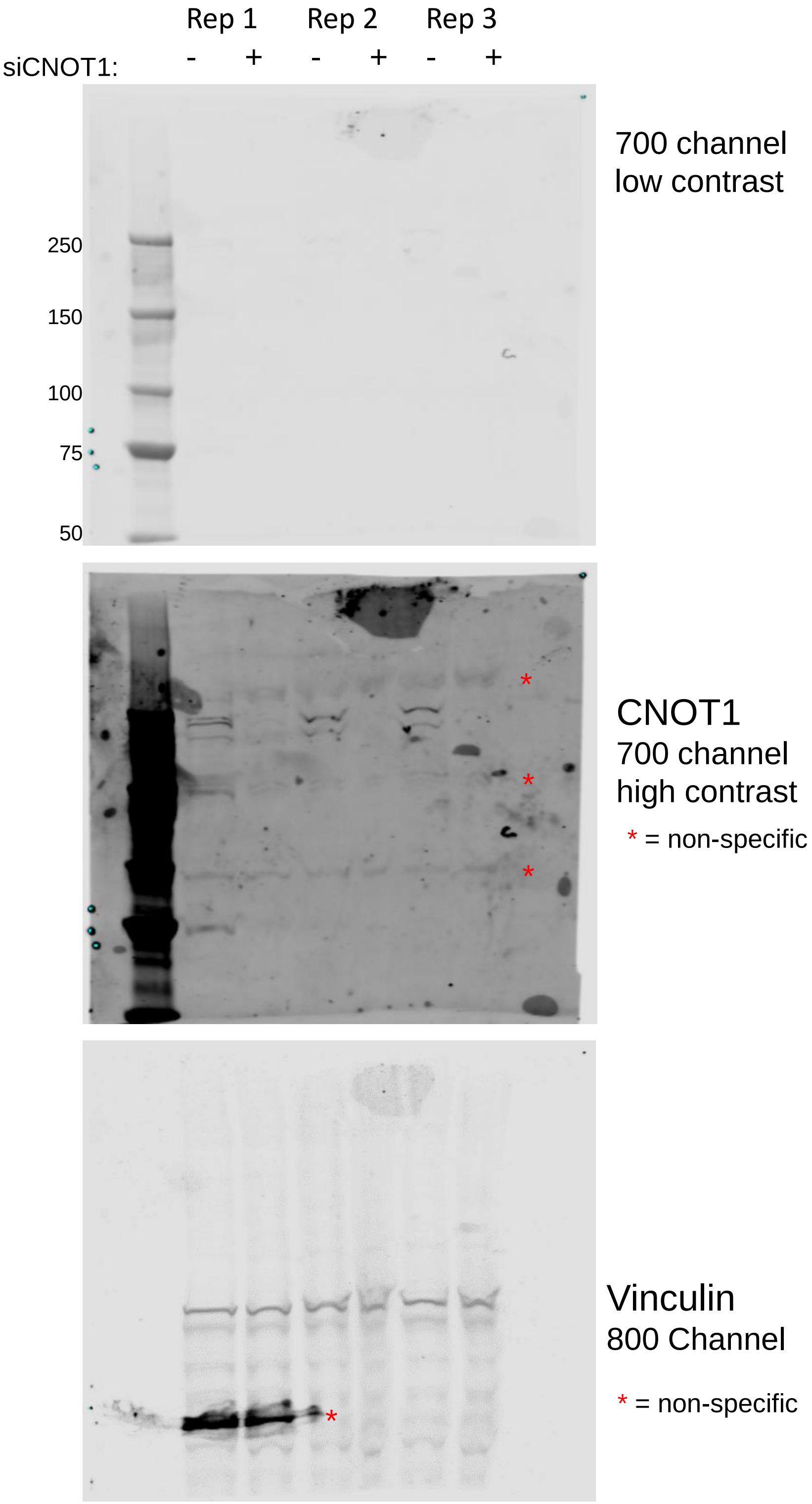

Additional File 3, Fig. S1: Uncropped western blot for Fig. 2A. Same membrane used for dual probing of CNOT1 and vinculin, with two different detection channels used.

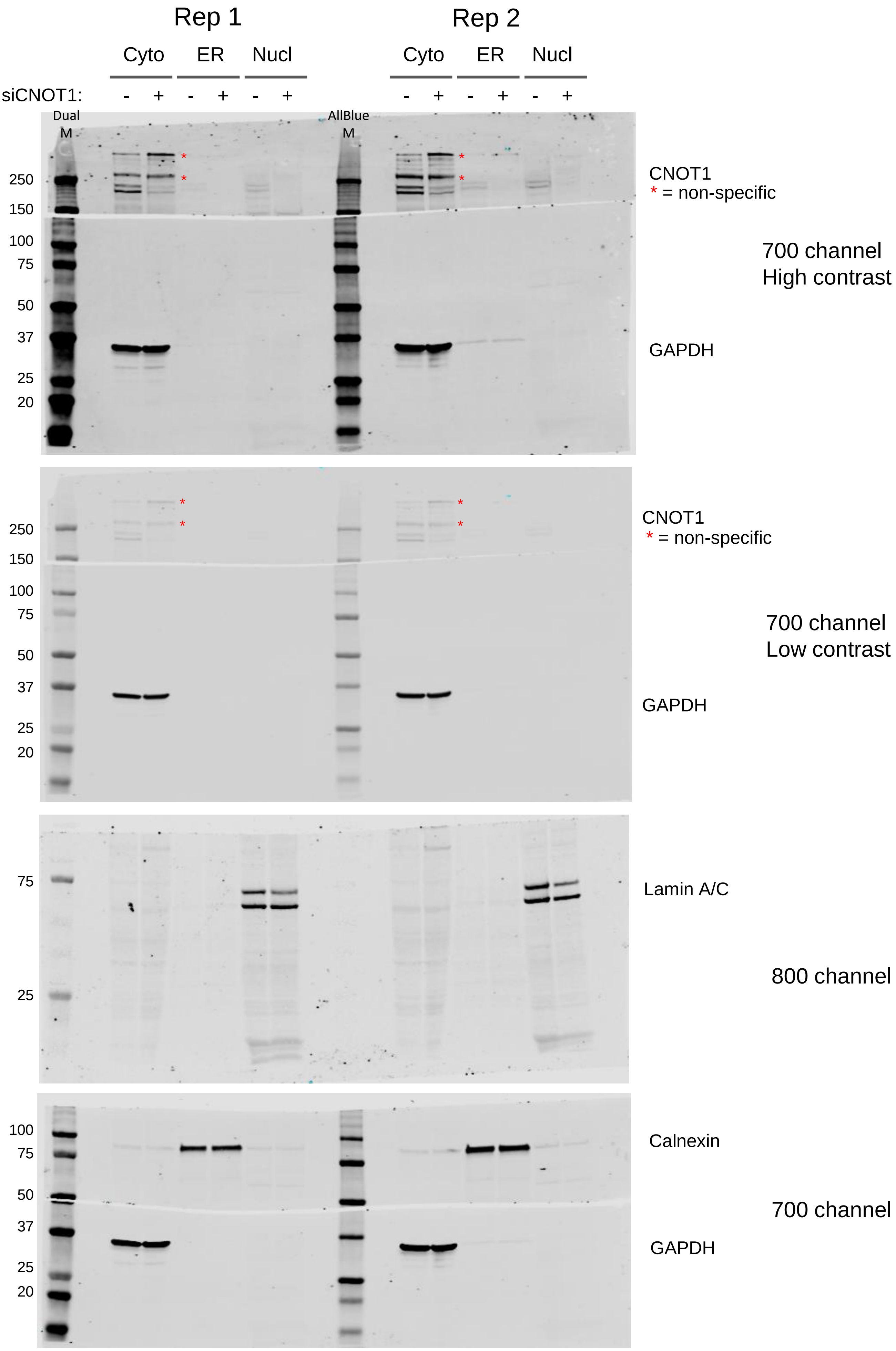

Additional File 3, Fig. S2: Uncropped western blot for Fig. 3B. Same membrane with parallel probing of CNOT1/GAPDH/LaminAC and sequential probing of Calnexin.

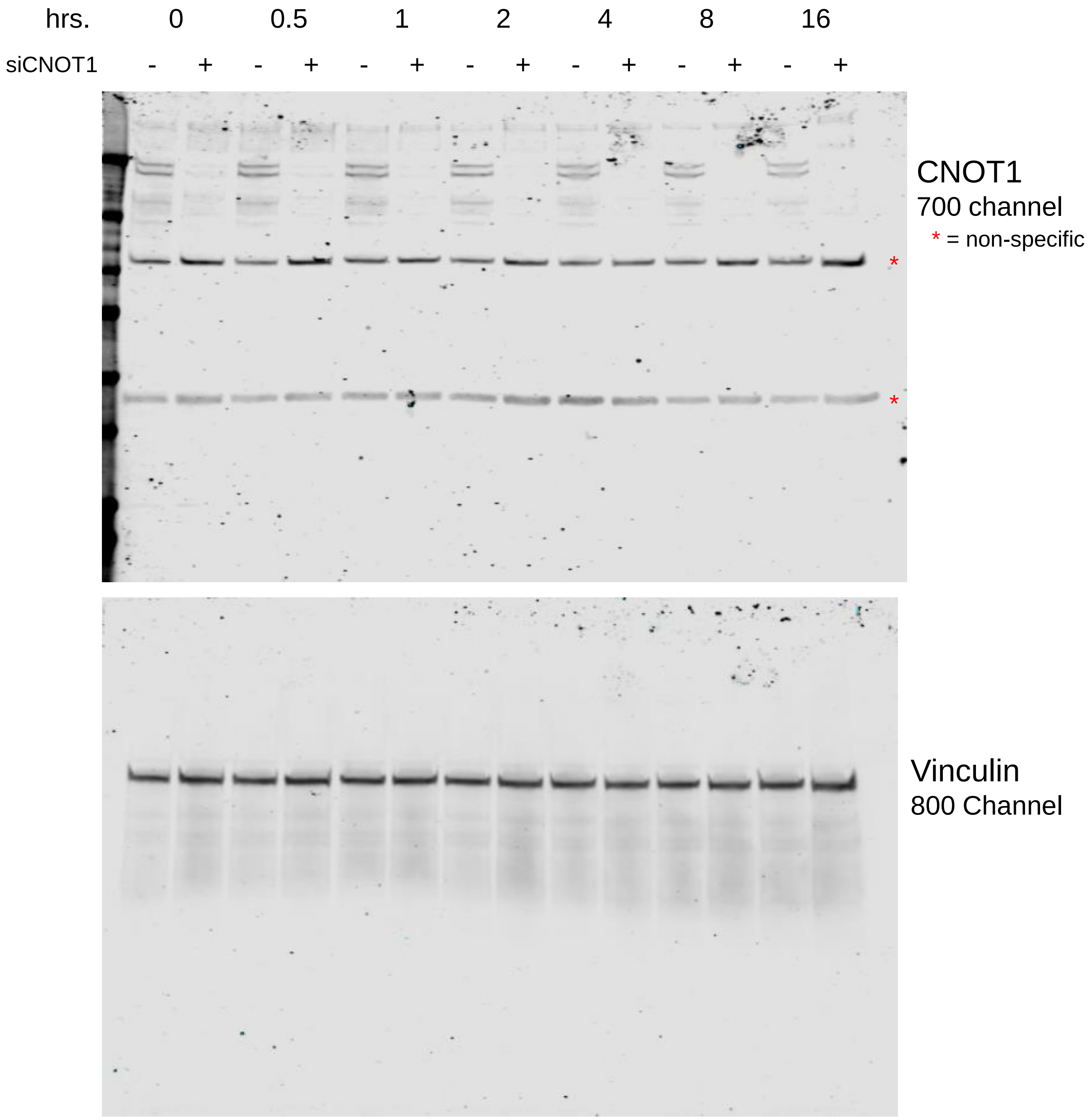

Additional File 3, Fig. S3: Uncropped western blot for Fig. S1E. Same membrane with sequential probing of CNOT1 followed by vinculin in two different detection channels.
