# Supplementary material for: Differential regulation of mRNA fate by the human Ccr4-Not complex is driven by coding sequence composition and mRNA localization
Source: Genome Biol. 2021 Oct 6;22:284. doi: 10.1186/s13059-021-02494-w (PMC8496106; doi:10.1186/s13059-021-02494-w)
Supplement: Supplementary file 11 — Additional file 11. Review history. [file 13059_2021_2494_MOESM11_ESM.docx]

Review History

**First round of review**

**Reviewer 1**

**Are you able to assess all statistics in the manuscript, including the appropriateness of statistical tests used?**

No, I do not feel adequately qualified to assess the statistics.

**Comments to author:**

GBIO-D-20-01716 - Differential regulation of mRNA fate by the human Ccr4-Not complex is driven by CDS composition and mRNA localisation

The Ccr4-Not complex is a conserved protein complex that regulates most steps during gene expression. Its most studied role is to facilitate the removal of the poly(A) tail and the destabilization of messenger RNAs in the cytoplasm. In addition, the Ccr4-Not also regulates transcription, mRNA export, translation and co-translation assembly of protein-complexes. Despite the wide range of functions attributed to the complex, a comprehensive understanding of the regulation of gene expression by this complex has not been done to date. Gillen et al., undertook a holistic approach and determined global changes in mRNA stability, translation efficiency and protein output in cells lacking CNOT1, the scaffold subunit of the complex. Their data suggests that in the human HEK293 cells the effects of the Ccr4-Not complex on mRNA stability and translation is mainly dictated by the nucleotide/codon composition of the coding sequence. This work provides a global view of the extent to which the Ccr4-Not complex influences gene expression in human cells. Furthermore, supported by several bioinformatic analysis, the authors identify nucleotide composition at the coding sequence (CDS) as a major determinant for the regulation of mRNA stability and translation of subsets of transcripts by the Ccr4-Not complex.

Despite the advance on how the Ccr4-Not may regulate different steps of gene expression, the majority of the observations and conclusions described in this work is not validated experimentally. This could be for example done with reporter transcript assays were the features described in the paper can be manipulated and tested for regulation by the deadenylation complex. In addition, some of the conclusions retrieved after bioinformatic analysis rely on weak correlations between the analyzed features.

Major and Specific points:

- Are the methods appropriate to the aims of the study, are they well described, and are necessary controls included? If not, please specify what is required.

The methods applied in this manuscript are appropriate to the questions raised in the study. In the majority of the cases, the methods are well described or referenced for further reading. The necessary controls have been included and analyzed.

One major concern associated with the methods applied in this work is related to the ribosome profiling experiments. According to the material and methods section, ribosome profiling was conducted as previously described in Wilczynska et al 2019. In the mentioned manuscript, cells were treated with cycloheximide for 3 minutes before harvesting. Pre-treatment of cells with cycloheximide prior to harvest is known to skew the natural distribution of elongating ribosomes, which can have considerable consequences for analyses that require single-codon resolution. Most recent ribosome-profiling experiments only include cycloheximide in the lysis buffer. Can the authors rule out that the conclusions retrieved from the ribosome profiling experiments are not influenced by pre-treating the cells with cycloheximide?

- Are the conclusions adequately supported by the data shown? If not, please explain

The majority of the conclusions presented in the manuscript are obtained by bioinformatic analysis of the data collected by RNA-Seq, Ribo-Seq and SILAC-proteomics. The retrieved observations and conclusions are sustained by statistical analysis and highlighted when significance is achieved. However, additional experimental data that tests the reported conclusions is absent in the majority of the cases.

Examples:

a)	The authors claim that GC-ending codons drive Ccr4-Not mediated mRNA destabilization. This observation can be tested with CDS-based reporter assays in which the sequence of the 3rd nucleotide of each codon is manipulated and tested for effects on mRNA stability in the presence and absence of CNOT1. These reporter assays can also be used to test the effects of CNOT1 depletion on translation efficiency and protein output. Additionally, the reporter assays can also be used to perform complementation experiments, in which addback of CNOT1 to cells is able to revert the observed effects upon depletion of the protein. This experiment was never performed for any of the features reported in the manuscript.

b)	The authors also state that depletion of CNOT1 decreases the translation efficiency of mRNAs associated with the endoplasmic reticulum (ER), extracellular matrix and plasma membrane. However, the authors do not corroborate this conclusion with the analysis of protein output for some mRNAs. This observation could be for instance supported by the analysis of the protein production determined by SILAC in this group of mRNAs compared to a control group. In addition, it is also unclear if CNOT1 depletion affects the secretory pathway. Indeed, some manuscripts report that disruption of the Ccr4-Not complex induces ER stress (Ito et al., 2011a; Ito et al., 2011b). It would be important to verify at least that the expression of signal recognition particle is not changing or ER-stress is not occurring in the conditions tested in the manuscript. Changes in the secretory pathway could influence the translation efficiency of specific mRNAs. The SILAC data also shows data several SRP components are less synthesized after CNOT1 knockdown. If the secretory pathway is inefficient, then CNOT1 primarily regulates components of this pathway.

c)	Another conclusion of this work is that the Ccr4-Not complex translationally regulates spatially localized groups of mRNAs (ER-associated or P-body localized). Such statement derives once more from pure bioinformatic analyses (Gene ontology and functional analysis or comparison with published data) and it is not backed up by experiments in cells that address mRNA localization in control conditions. As the authors state, it is known that depletion of CNOT1 prevents P-body formation. Thus, translational upregulation of P-body enriched mRNAs in the absence of CNOT1 might be caused by the effects on the assembly of these RNA granules, and not a direct role of the Ccr4-Not complex in the repression of translation of specific mRNAs.

d)	The experiments described in the manuscript also do not address if the observed effects are associated with the full deadenylation complex, or specifically with CNOT1. Fig. 1A should also show the expression of other subunits of the complex (CNOT1 depletion is known to affect the expression of the full complex) or supported by the protein output for the components of the complex estimated by SILAC-proteomics.

e)	The authors observed a strong correlation between ribosome occupancy and protein output. They also state that they also observe several mRNAs that do not follow this trend. Could the authors provide an estimation of how many mRNAs belong to such group and which type of effect was observed? As the manuscript does not contemplate the effects of CNOT1 knockdown in protein stability it could be interesting to see how many and which mRNAs can undergo post-translational regulation in these conditions. In figure 6A, it would be important also to highlight the groups of genes with fundamental changes in RNA levels and TE in the absence of CNOT1 (eg.: ER-targeted mRNAs, nuclear proteins, zinc finger proteins…). This information could help to understand how changes in RNA and TE upon CNOT1 knockdown impact protein output.

f)	Experimental testing of the features described in the manuscript that rely on weak correlations would be crucial to support such findings (eg.: correlation of A/U or G/C ending codons with log2FC TE or protein production).

g)	The authors also claim that the Ccr4-Not complex can regulate ribosome pausing in human cells. Is this a widespread effect? Is it related with RNA abundance, TE or protein output? Only a few examples are presented in the manuscript. Can the authors elucidate the number of mRNAs with increased/ decreased pausing in the absence of CNOT1? Is there a preference for codon, amino acid or motif at the pause sites?

h)	Another conclusion from the analyzed data is that nuclear proteins (enriched for transcriptional factors) are translationally upregulated following CNOT1 depletion. Can the authors comment if they observe changes in overall transcription? This information would be relevant as the deadenylation complex has also been shown to regulate transcription.

- Are sufficient details provided to allow replication and comparison with related analyses that may have been performed? If not, please specify what is required.

The details provided in the material and methods section are sufficient to allow replication and comparison with related analyses.

- Does the work represent a significant advance over previously published studies?

This work provides new information on the genome-wide effects of the depletion of CNOT1 in human cells, with focus on changes in mRNA levels, translation efficiency and protein output. As such data has not been so far available to the research community, the manuscript represents a significant of advance in the literature.

- Is the paper of broad interest to others in the field, or of outstanding interest to a broad audience of biologists?

Based on the broad and crucial roles of the Ccr4-Not in physiological and pathological conditions, this manuscript is of interest to the fields of RNA and Translation, but also to all biologists interested in the mechanisms underlying the regulation of gene expression in human cells.

Minor points:

The codon labels in Figure 5A are not clear (too blurry).

References:

Ito, K., Inoue, T., Yokoyama, K., Morita, M., Suzuki, T., and Yamamoto, T. (2011a). CNOT2 depletion disrupts and inhibits the CCR4-NOT deadenylase complex and induces apoptotic cell death. Genes Cells 16, 368-379.

Ito, K., Takahashi, A., Morita, M., Suzuki, T., and Yamamoto, T. (2011b). The role of the CNOT1 subunit of the CCR4-NOT complex in mRNA deadenylation and cell viability. Protein Cell 2, 755-763.

**Reviewer 2**

**Are you able to assess all statistics in the manuscript, including the appropriateness of statistical tests used?**

No, I do not feel adequately qualified to assess the statistics.

**Comments to author:**

The manuscript describes the effects of NOT1 knock-down in combination with drug treatment (triptolide and cyclohemixide) for effects on mRNA expression, mRNA ribosome occupancy and translational rates using RNA-seq, ribosome profiling and MS-based proteomics for a system-wide analysis.

The authors here combine several omics technologies, which is a technical and analytical challenge and while some of their data looks very intriguing (e.g. signature of codon usage in Fig. 1E), the manuscript needs to be severely strengthened experimentally and for statistical analysis.

Conceptional:

1)	I have a major issue with the authors claiming to have studied CCR4-NOT complex, while the data was obtained with a siCNOT1 knockdown using 1 single siRNA (Ambion S22844). To even evaluate potential off target effects, at least a second independent siRNA should be used nowadays.

2)	They always claim to have studied the function of the CCR-NOT1 complex, but only targeted a single subunit CNOT1. While this destabilizes the complex and may impair functionality of the remaining subcomplexes, this is neither shown nor discussed. It may be some functionality is still conserved while some other failed. However, this may change several of their conclusions as the molecular phenotypes may arise from uncoordinated action of the individual (sub-)complexes without the docking platform CNOT1.

3)	Treatments with triptolide and cycloheximide (known to have codon preferences in ribosome profiling!) can have individual effects when combined with siRNA knockdown. Here, it would have been crucial to use a second drug to delineate possible drug-specific effects from a global gene regulatory picture.

Experimental:

1)	The authors use siRNA for knockdown. I don't necessarily have a problem with the split blots in Figure S1D, but it is not benefical that the exposure or developing seems not to have been done in parallel. This is a time-course experiment. Unfortunately, can't be changed retrospectively, but in the future the blots should be done in parallel.

2)	Ribosome profiling was conducted 48 hours past siRNA treatment while pSILAC was started at 30 hours past induction and harvest 14 hours later (44 hours). It is a little unclear why proteome analysis was conducted timely prior to translational analysis. Actually, I think both should have been time course analysis as well or at least conducted at 2 time points to see whether the results are stable or time-dependent after siRNA. (I would suggest they are time-dependent as the RNA level changes shown in Fig. 1A, however then the time is a confounding factor in their analysis.)

3)	The hypothesis of changes in subcellular localization changes should at least be attempted to be tested by RNA-FISH experiments. They are doable with a couple candidates in a miniscreen.

4)	I am not so inclined to follow the authors on speculation of compensation for mRNA stability on protein level as no correlation between mRNA and protein level for the same time point or a few hours later (delay in translation) was shown, but protein measurements are done earlier than ribosome profiling.

Stastistical:

1)	I don't really see the benefit of gradient boosting for the method of choice to perform subsequent feature detection in their dataset. Could the authors please explain why this approach should be suitable for several of their datatypes? Or in other words, on what has it been trained?

2)	The application of the caret R package needs to be described in more detail in the methods. Made assumptions, used variables, etc…

4)	For Figure 1B, a clustering algorithm combined with stating how many different clusters and the cluster size (e.g k-means) would be a more mathematical analysis of the data than showing examples. It would also tell more about how prevalent certain behaviors are.

5)	Was mRNA abundance considered as a confounding factor in mRNA stability or translation rate (i.e. was it considered as a feature in gradient boosting?). If not, why not?

Minor:

1)	Result part: overstatement, I would not call this a "holistic" approach as the study.

2)	GC-ending codons: "here we expand this on showing how amino acid differences contribute to the nature of impact of GC/AU ending codons in mRNA stability." That is not shown and the sentence is not logical.

3)	Why do the authors use violin plots (Figure 4B) and boxplots (Figure 4D-F) for represent similar data? In case there is no specific reason, could this be harmonized throughout all figures?

4)	I would not hide the statistics for the figures in the supplementary. Just to illustrate on Figure 4B, the reader is left with the distributions, but while they all look different by eye (maybe), only some are statistical significant. One could think about a mini-comparion like did in the Additional File2, putting it on the right to the figure, not with the long (overaccurate numbers, but with color when statististical significant.

5)	The number of proteins in the bins in 4B is not the number of mRNAs in Fig. 4A. Could the authors state the size of the bins in Figure 4B either in the Table of Figure 4A or in 4B again.

6)	u should be correct for µ

Requested comments:

The pSILAC experiment seems ok. Suppl. Table 4 should not just contain the gene names, but also the unique identifiers such as Uniprot or whatever was used for the MQ search. Especially as MQ output results in proteinGroups. Additionally, it would be helpful to have the proteinGroups intensity data in this table as well.

**Authors Response**

**Point-by-point responses to the reviewers’ comments:**

Reviewer #1

*We thank the reviewer for their comprehensive and insightful review. We are pleased the reviewer finds our “manuscript represents a significant advance in the literature”.*

*Below we address the reviewer’s queries and suggestions point-by-point:*

Major and Specific points:

1. One major concern associated with the methods applied in this work is related to the ribosome profiling experiments. According to the material and methods section, ribosome profiling was conducted as previously described in Wilczynska et al 2019. In the mentioned manuscript, cells were treated with cycloheximide for 3 minutes before harvesting. Pre-treatment of cells with cycloheximide prior to harvest is known to skew the natural distribution of elongating ribosomes, which can have considerable consequences for analyses that require single-codon resolution. Most recent ribosome-profiling experiments only include cycloheximide in the lysis buffer. Can the authors rule out that the conclusions retrieved from the ribosome profiling experiments are not influenced by pre-treating the cells with cycloheximide?

*Although potential codon-level biases have been observed, it is important to note that the cycloheximide bias has largely been studied in yeast (Duncan et al, 2017; Hussmann et al, 2015; Santos et al, 2019), and a follow up study has shown that the cycloheximide induced bias at single-codon resolution is species-specific and was not apparent in human cells (Sharma et al, 2019). In mouse embryonic stem cells, it was shown that the primary difference in pre-treatment with cycloheximide is limited to the AUG start codon, 5-10 codons downstream and the stop codon (Ingolia et al, 2011). Most of our ribo-seq analyses are based on CDS sequences with the first 15 and last 5 codons excluded, to specifically avoid these issues. Also, our analysis focuses on the differential between CNOT1 knockdown and control condition which should ‘normalise out’ cycloheximide-specific effects and therefore not impact out results. In addition, the qPCR validations we included (Fig.1DEF, Additional File 1: Fig. S6) for the translational efficiency groups were conducted from independent experiments with no cycloheximide pre-treatment to ensure our results were not confounded by this.*

However, additional experimental data that tests the reported conclusions is absent in the majority of the cases.

Examples:

a) The authors claim that GC-ending codons drive Ccr4-Not mediated mRNA destabilization. This observation can be tested with CDS-based reporter assays in which the sequence of the 3rd nucleotide of each codon is manipulated and tested for effects on mRNA stability in the presence and absence of CNOT1. These reporter assays can also be used to test the effects of CNOT1 depletion on translation efficiency and protein output. Additionally, the reporter assays can also be used to perform complementation experiments, in which addback of CNOT1 to cells is able to revert the observed effects upon depletion of the protein. This experiment was never performed for any of the features reported in the manuscript.

*We have now designed reporters to address this. Given that the Renilla luciferase sequence is rich in A/U-ending codons (74.5%) we have designed reporters in which we have increased the frequency of the G/C-ending codons. The codons identified as most highly correlated with a reduction in translational efficiency following CNOT1 knockdown (AUC/GUC/ACC, Additional File 1: Fig. S9A) were substituted for the synonymous codons in the Renilla CDS so as not to impact the amino acid composition. By way of luciferase activity for protein levels and qPCR for the luciferase RNA levels the translational efficiency of these reporters was examined. We observe that converting these codons is sufficient to reduce the translational efficiency change occurring with CNOT1 knockdown compared to the original Renilla sequence (Additional File 1: Fig. S9B). Also, given reviewer 2 conceptional point 1, we have conducted this reporter assay with an additional siRNA pool targeting CNOT1, which confirms this result is siRNA independent.*

*Add-back experiments would be extremely difficult to perform given the size of the CNOT1 protein and the likely stress on cells caused by simultaneous knockdown and overexpression. Nevertheless, we are confident that the results are specific to CNOT1, having validated specific findings using different siRNAs (Additional File 1 Fig. S2DEF & Fig. S9B).*

b) The authors also state that depletion of CNOT1 decreases the translation efficiency of mRNAs associated with the endoplasmic reticulum (ER), extracellular matrix and plasma membrane. However, the authors do not corroborate this conclusion with the analysis of protein output for some mRNAs. This observation could be for instance supported by the analysis of the protein production determined by SILAC in this group of mRNAs compared to a control group.

*We thank the reviewer for pointing out the lack of this data. In response to experimental comment 3 from reviewer 2 we have now determined mRNAs preferentially localised to the ER and cytosol in our exact cells (Fig. 3BC). We then compared the protein output of ER-targeted mRNAs with cytosolic mRNAs in the pulsed SILAC data and indeed observe reduced protein synthesis (Fig. 3I) which suggests the reduced translational efficiency also results in reduced protein output from these mRNAs after CNOT1 depletion.*

*In addition, it is also unclear if CNOT1 depletion affects the secretory pathway. Indeed, some manuscripts report that disruption of the Ccr4-Not complex induces ER stress (Ito et al., 2011a; Ito et al., 2011b). It would be important to verify at least that the expression of signal recognition particle is not changing or ER-stress is not occurring in the conditions tested in the manuscript. Changes in the secretory pathway could influence the translation efficiency of specific mRNAs. The SILAC data also shows data several SRP components are less synthesized after CNOT1 knockdown. If the secretory pathway is inefficient, then CNOT1 primarily regulates components of this pathway.*

*While the pulsed SILAC shows a slight decrease in synthesis of the SRP proteins, at 48hrs we do not see a decrease in the overall protein level of several SRP proteins by western blot:*

*This suggests reduced SRP expression is not the cause of the reduced translational efficiency observed on ER-targeted mRNAs.*

*We have also checked for ER-stress by looking at p-eIF2α levels, using thapsigargin (TSG) as a positive control for ER-stress induction and we do not observe a change in the p-eIF2α / total eIF2α levels with CNOT1 knockdown. This apparent difference between the published study (Ito et al, 2011) and our work may be a result of the different cell line used and their later time point of 72 hrs after CNOT1 depletion.*

*In addition, to address reviewer 2 experimental comment 3 we have carried out fractionation experiments -/+ siCNOT1 and sequenced the mRNA in the ER and cytosolic fractions. We observe that mRNAs that are predominantly ER-associated have reduced levels specifically in the ER following CNOT1 depletion (Fig. 3H), supporting the hypothesis that CNOT1 knockdown leads to a reduction in the proportion of mRNAs localising to the ER. This new data is presented in Fig. 3BCD, 3FGHI.*

c) Another conclusion of this work is that the Ccr4-Not complex translationally regulates spatially localized groups of mRNAs (ER-associated or P-body localized). Such statement derives once more from pure bioinformatic analyses (Gene ontology and functional analysis or comparison with published data) and it is not backed up by experiments in cells that address mRNA localization in control conditions. As the authors state, it is known that depletion of CNOT1 prevents P-body formation. Thus, translational upregulation of P-body enriched mRNAs in the absence of CNOT1 might be caused by the effects on the assembly of these RNA granules, and not a direct role of the Ccr4-Not complex in the repression of translation of specific mRNAs.

*As detailed in response to comment b above and to reviewer 2 experimental point 3 we have now conducted fractionation experiments to assess the distribution of mRNAs localised in the cytoplasm and the ER and see that this population of mRNAs has reduced distribution to the ER (new data in Fig. 3).*

*In addition, we have now added to the discussion to make it clear that the effects of CNOT1 depletion on p-body enriched mRNAs could be due to the role of CNOT1 in the formation of p-bodies as opposed to directly acting upon the mRNAs themselves.*

*The text reads: ‘In addition, our data shows a role for the Ccr4-Not complex in the translational repression of mRNAs that are localised to p-bodies (Additional File 1: Fig. S9C). It will be interesting to investigate in the future whether this is due to an indirect role of CNOT1 in p-body formation (Ito et al, 2011) or if the Ccr4-Not complex is also involved in mediating repression that occurs within these granules.’*

d) The experiments described in the manuscript also do not address if the observed effects are associated with the full deadenylation complex, or specifically with CNOT1. Fig. 1A should also show the expression of other subunits of the complex (CNOT1 depletion is known to affect the expression of the full complex) or supported by the protein output for the components of the complex estimated by SILAC-proteomics.

*We have now added a table (Additional File 1: Fig. S1A) highlighting the changes in all Ccr4-Not complex subunit proteins in the pulsed SILAC following CNOT1 depletion. In agreement with previously published data (Ito et al, Protein Cell 2011) we see the reduced synthesis of several of the Ccr4-Not subunits following CNOT1 depletion. Combined with existing data about how the activity of the Ccr4-Not complex is dependent on the presence of CNOT1 (Ito et al, 2011, Raisch et al, 2018), this suggests that the depletion of CNOT1 results in the abrogation of function of the whole Ccr4-Not complex in our experiments. We have now added to the text to make this clearer: ‘Knockdown of CNOT1 also downregulates the synthesis of many other subunits of the Ccr4-Not complex (Ito et al, 2011), which our results confirm (Additional File 1: Fig. S1A)’.*

e) The authors observed a strong correlation between ribosome occupancy and protein output. They also state that they also observe several mRNAs that do not follow this trend. Could the authors provide an estimation of how many mRNAs belong to such group and which type of effect was observed? As the manuscript does not contemplate the effects of CNOT1 knockdown in protein stability it could be interesting to see how many and which mRNAs can undergo post-translational regulation in these conditions. In figure 6A, it would be important also to highlight the groups of genes with fundamental changes in RNA levels and TE in the absence of CNOT1 (eg.: ER-targeted mRNAs, nuclear proteins, zinc finger proteins…). This information could help to understand how changes in RNA and TE upon CNOT1 knockdown impact protein output.

*Please see the response to comment g as we have now improved and updated the analysis previously presented in Fig. 6.*

*We agree that there is a possibility that protein stability changes may occur in the absence of CNOT1, however as our objective was to understand the effect of its depletion on the core activity of the Ccr4-Not complex in regulating mRNA translation and turnover, we focused on assays that would directly address these questions. Investigation of protein stability would be an interesting avenue for future investigations.*

*Fig. 4B depicts protein production changes for groups differentially regulated in terms on translational efficiency and mRNA stability, which clearly shows how changes in mRNA stability and translational efficiency following CNOT1 knockdown are reflected in protein production changes. In addition, in response to comment b we have now added Fig. 3I that specifically looks at the impact of CNOT1 knockdown on the protein production from ER-target mRNAs.*

f) Experimental testing of the features described in the manuscript that rely on weak correlations would be crucial to support such findings (eg.: correlation of A/U or G/C ending codons with log2FC TE or protein production).

*The reviewer is correct in pointing this out. In fact, the plot showing correlations between the A/U or G/C ending codons and protein production was intended to demonstrate that there is no apparent correlation between the third position of the codon and protein production. We have now modified the text to make this point clearer ‘Further investigation of how codon frequencies ultimately impact protein synthesis demonstrates that although the 3rd nucleotide preference of codons contributes to mRNA stability and translational efficiency regulated by the Ccr4-Not complex (Fig.1F & Additional File 1: Fig. S9A), their frequency does not strongly correlate with protein production changes after CNOT1 depletion (Additional File 1: Fig.S9D)’.*

*As for the correlations with the log2FC TE, there is a clear separation in third position nucleotide between mRNAs that have higher and lower translational efficiency after CNOT1 depletion. As detailed in response to comment a, we have now shown that changing A/U-ending codons in Renilla to the synonymous G/C-ending codons leads to a reduction in the translational efficiency change occurring with CNOT1 depletion compared to the original Renilla sequence (Additional File 1: Fig. S9B).*

g) The authors also claim that the Ccr4-Not complex can regulate ribosome pausing in human cells. Is this a widespread effect? Is it related with RNA abundance, TE or protein output? Only a few examples are presented in the manuscript. Can the authors elucidate the number of mRNAs with increased/ decreased pausing in the absence of CNOT1? Is there a preference for codon, amino acid or motif at the pause sites?

*To fully address these questions, we have now conducted a more comprehensive analysis of ribosome pause sites. First, a pause site in each condition was defined as a position with a RPF (ribosome protected fragment) peak height ten times greater than the average RPF peak on the mRNA. Second, these pause sites were then classified as either ‘sustained’ meaning they are present but unaltered with CNOT1 knockdown; ‘resolved’ in that the reduction in RPF peak height is ten times greater than the average delta decrease across the mRNA or ‘induced’ in that the increase in RPF peak height is ten times greater than the average delta increase. The number of mRNAs that contain peaks of these types are shown in the Venn diagram in Fig. 6A.*

*We show that the presence or induction of pause sites upon CNOT1 knockdown is associated with greater stabilisation of mRNAs (Fig. 6E) and that resolution of pause sites by CNOT1 depletion leads to increased protein production relative to the change in ribosome residency (Fig. 6F).*

*Finally, we have now examined the sequences at these pause sites, and we find an enrichment for charged amino acids at the A-site of paused ribosomes that are sustained or resolved in the absence of CNOT1 (Fig. 6GH).*

h) Another conclusion from the analyzed data is that nuclear proteins (enriched for transcriptional factors) are translationally upregulated following CNOT1 depletion. Can the authors comment if they observe changes in overall transcription? This information would be relevant as the deadenylation complex has also been shown to regulate transcription.

*Indeed, we cannot exclude the possibility that transcription is affected by depletion of CNOT1. The methodologies used in our work do not allow us to assess this, and we anticipate that manipulation of transcription on a global scale for a high throughput analysis could lead to cellular toxicity. As mentioned above, our study was focused on the complex’s post-transcriptional activities.*

Minor points:

The codon labels in Figure 5A are not clear (too blurry).

*This has now been corrected.*

Reviewer #2

Conceptional:

1) I have a major issue with the authors claiming to have studied CCR4-NOT complex, while the data was obtained with a siCNOT1 knockdown using 1 single siRNA (Ambion S22844). To even evaluate potential off target effects, at least a second independent siRNA should be used nowadays.

*In experiments addressing comment 3 below, we have now validated the mRNA stability determined by the RNA-sequencing data using an additional transcriptional inhibitor - flavopiridol. We have also done this using an additional siRNA pool targeting CNOT1 (dharmacon pool of 4 siRNAs) and by qPCR clearly show the results are reproducible with this different set of siRNAs targeting CNOT1 (Fig. S2DEF). Also, in response to reviewer 1 comments a & f we have conducted reporter experiments, and the result is consistent when using both our original siRNA and the additional siRNA pool targeting CNOT1.*

*Thus, the effects we observe are siRNA independent.*

2) They always claim to have studied the function of the CCR-NOT1 complex, but only targeted a single subunit CNOT1. While this destabilizes the complex and may impair functionality of the remaining subcomplexes, this is neither shown nor discussed. It may be some functionality is still conserved while some other failed. However, this may change several of their conclusions as the molecular phenotypes may arise from uncoordinated action of the individual (sub-)complexes without the docking platform CNOT1.

*It has previously been reported that CNOT1 knockdown results in reduction in the levels of several other components of the Ccr4-Not complex (Ito et al, 2011). We have now added a table (Additional File 1: Fig. S1A) highlighting the changes in all Ccr4-Not complex subunit proteins in the pulsed SILAC following CNOT1 depletion. As shown previously (Ito et al, 2011), we see the reduced synthesis of several of the Ccr4-Not subunits following CNOT1 depletion. Combined with existing data about how the activity of the Ccr4-Not complex is dependent on the presence of CNOT1 (Ito et al, 2011, Raisch et al, 2018), this suggests that the depletion of CNOT1 results in the abrogation of function of the whole Ccr4-Not complex in our experiments.*

3) Treatments with triptolide and cycloheximide (known to have codon preferences in ribosome profiling!) can have individual effects when combined with siRNA knockdown. Here, it would have been crucial to use a second drug to delineate possible drug-specific effects from a global gene regulatory picture.

*Triptolide was specifically chosen because it is not known to have sequence bias – unlike some transcriptional inhibitors such as actinomycin D (Bensaude, 2011). Nevertheless, we have now validated the mRNA half-lives determined by the RNA-sequencing data using an additional transcriptional inhibitor (flavopiridol) and qPCR analysis clearly shows the results are reproducible (Fig. S2DEF) and thus the effects we observe are drug independent. Considering comment 1, we have done these flavopiridol experiments with 2 different siRNAs targeting CNOT1.*

*We have made the appropriate adjustments in the text to reflect this additional data.*

*For ribosome profiling cycloheximide is specifically used as a tool to obtain ribosome protected fragments – in this context there is not an appropriate second drug. Although potential codon-level biases have been observed, it is important to note that the cycloheximide bias has largely been studied in yeast (Duncan et al, 2017, Hussmann et al, 2015, Santos et al, 2019), and a follow up study has shown that the cycloheximide induced bias at single-codon resolution is species-specific and was not apparent in human cells (Sharma et al, 2019). In fact, previous findings from mammalian ribosome profiling experiments have not been contested in the literature because of cycloheximide pre-treatment, while ones carried out in yeast have.*

*Nevertheless, the qPCRs we included (Fig. 2DEF, Additional File 1: Fig. S6) for the validation of the translational efficiency groups were conducted from independent experiments with no cycloheximide pre-treatment to ensure our results were not confounded by this. Also, our analysis focuses on the differential between CNOT1 knockdown and control condition which should ‘normalise out’ cycloheximide specific effects. There is no evidence or reason to suspect siRNAs causing specific effects in combination with cycloheximide.*

Experimental:

1) The authors use siRNA for knockdown. I don't necessarily have a problem with the split blots in Figure S1D, but it is not benefical that the exposure or developing seems not to have been done in parallel. This is a time-course experiment. Unfortunately, can't be changed retrospectively, but in the future the blots should be done in parallel.

*We have now purchased gels with a greater number of wells and have rerun the gels with the remaining lysate. The figure in the manuscript has now been replaced (Fig. S1E).*

2) Ribosome profiling was conducted 48 hours past siRNA treatment while pSILAC was started at 30 hours past induction and harvest 14 hours later (44 hours). It is a little unclear why proteome analysis was conducted timely prior to translational analysis. Actually, I think both should have been time course analysis as well or at least conducted at 2 time points to see whether the results are stable or time-dependent after siRNA. (I would suggest they are time-dependent as the RNA level changes shown in Fig. 1A, however then the time is a confounding factor in their analysis.)

*Fig. 1A shows a time course following the additional of a transcriptional inhibitor. In order to assess mRNA half-life, a time-course must be conducted.*

*Previous experiments have shown us that harvesting after 48h of CNOT1 depletion strikes the best balance between attaining a steady-state knockdown and mitigating the adverse effects of transfection of the siRNAs. This is why the harvest time for experiments not requiring more than one time point was chosen to be around 48hrs. Whereas, pulsed SILAC is a methodology that requires a pulse of labelled amino acids to be administered on the cells for an extended number of hours. Our pulsed SILAC clearly demonstrates that CNOT1 is the most downregulated protein in the analysis – serving effectively as an internal control for the experiment. The same is true for our ribosome profiling and RNA-seq data. As our scientific question was focused on the steady-state effects of depletion of CNOT1, none of these techniques required multiple time points.*

3) The hypothesis of changes in subcellular localization changes should at least be attempted to be tested by RNA-FISH experiments. They are doable with a couple candidates in a miniscreen.

*This is an excellent point, however, since the effect we see impacts a large group of mRNAs, we chose to address this in a more comprehensive manner by subcellular fractionation experiments (with and without CNOT1 knockdown) followed by RNA-seq (Fig. 3BC). With the use of spike-ins and analysis of the relative abundance of mRNAs in the ER and cytosolic fractions using unsupervised k-means clustering we clearly identify 2 clusters of mRNAs in control conditions, one of which we assign as ER-target mRNAs and the other as predominantly localised in the cytosol (Fig. 3C). First, we have updated Fig. 3D to show the enrichment for ER-target mRNAs in the group of mRNAs with decreased TE following CNOT1 depletion is present in our own new dataset – with this new dataset the proportion identified as ER-target mRNAs is even greater than identified previously.*

*Next, we have assessed how the localisation of these ER-targeted and cytosolic mRNAs is altered following CNOT1 knockdown. This shows that specifically the ER-targeted mRNAs have reduced mRNA-levels in the ER fraction compared to mRNAs that are predominantly localised to the cytosol in control conditions (Fig. 3GH).*

*Moreover, using our pulsed SILAC data with these two groups of mRNAs, we further see that our observations of reduced TE on ER-target mRNAs is reflected in reduced protein synthesis of these messages (Fig. 3I).*

*The text has been altered to include this additional data.*

4) I am not so inclined to follow the authors on speculation of compensation for mRNA stability on protein level as no correlation between mRNA and protein level for the same time point or a few hours later (delay in translation) was shown, but protein measurements are done earlier than ribosome profiling.

*Translation is a very fast process and the time point at which the assays were done is well within a steady-state situation. We were referring here to the theory that a degree of translational buffering occurs in gene expression (Timmers et al, 2018). However, we agree that since we cannot provide any data to either prove or disprove this, we have removed the comment about buffering from the manuscript.*

Stastistical:

1) I don't really see the benefit of gradient boosting for the method of choice to perform subsequent feature detection in their dataset. Could the authors please explain why this approach should be suitable for several of their datatypes? Or in other words, on what has it been trained?

*We were interested in understanding what variables (i.e. characteristics of the mRNA) could be determined to have the highest influence on the observed results of CNOT1 on mRNA stability and translational efficiency. Gradient boosting is specifically useful for this purpose as it is a method from which feature importance scores can easily be obtained. This was conducted with 10-fold cross validation which randomly splits the data into 10 groups and is repeated using each fold as the test data set and the remainder as the training set. The successful implementation of this is not only shown by the downstream codon usage analysis but is now also further supported with reporter assays that show changing the A/U ending codons to synonymous G/C ending codons leads to a reduction in the translational efficiency change of Renilla luciferase after CNOT1 depletion (Additional File 1: Fig. S9B).*

2) The application of the caret R package needs to be described in more detail in the methods. Made assumptions, used variables, etc…

*We have now updated the methods. All the variables used in the analysis are shown in Additional File 1: Fig. S3B. For Fig. 1D a gaussian distribution was assumed and for Fig. 4C a multinomial distribution. The parameters used were: n.trees = 200, interaction.depth = 6, shrinkage = 0.005, cv.folds = 10. These details have now been added to the materials and methods.*

4) For Figure 1B, a clustering algorithm combined with stating how many different clusters and the cluster size (e.g k-means) would be a more mathematical analysis of the data than showing examples. It would also tell more about how prevalent certain behaviors are.

*Thank you for this suggestion, we have now used k-means clustering and identified 3 major clusters that differentiate the half-lives of the mRNAs in the presence and absence of CNOT1 (Fig. 1B). We have also added Fig. 1C to clearly depict how the mRNA half-life in the presence and absence of CNOT1 differentiates these 3 groups. Fig. S2ABC now show specific example mRNAs from each cluster from the sequencing data. Alongside this, in response to conceptional points 1 & 3, we have also included qPCR validation of the mRNA half-life of these example mRNAs from each cluster with a second transcriptional inhibitor and additional CNOT1-targeting siRNA pool (Fig. S2DEF).*

5) Was mRNA abundance considered as a confounding factor in mRNA stability or translation rate (i.e. was it considered as a feature in gradient boosting?). If not, why not?

*mRNA abundance was not considered as a factor in the feature analysis, because this analysis was based on calculations of mRNA stability and translation rate changes, which by definition incorporate RNA abundance. Our investigation was specifically mRNA sequence centric and therefore this feature analysis was used to identify cis regulatory elements on the mRNA that may impact how the Ccr4-Not complex regulates mRNA fate. For example, this analysis identified GC content of the CDS as an important feature, which we have now investigated further using reporter assays which validate this finding.*

Minor:

1) Result part: overstatement, I would not call this a "holistic" approach as the study.

*We have now changed the word ‘holistic’ in the text to ‘comprehensive’.*

2) GC-ending codons: "here we expand this on showing how amino acid differences contribute to the nature of impact of GC/AU ending codons in mRNA stability." That is not shown and the sentence is not logical.

*This has now been rephrased: ‘Here we expand on this showing how amino acid differences contribute to the nature of the impact of G/C or A/U ending codons on mRNA stability’.*

3) Why do the authors use violin plots (Figure 4B) and boxplots (Figure 4D-F) for represent similar data? In case there is no specific reason, could this be harmonized throughout all figures?

*Figure 4B has now been changed to a boxplot.*

4) I would not hide the statistics for the figures in the supplementary. Just to illustrate on Figure 4B, the reader is left with the distributions, but while they all look different by eye (maybe), only some are statistical significant. One could think about a mini-comparion like did in the Additional File2, putting it on the right to the figure, not with the long (overaccurate numbers, but with color when statististical significant.

*It was certainly not our intention to hide any statistics. They were simply included in the supplemental file to ease presentation of the statistics for the multiple comparisons. To address this reviewer’s concern and for ease of plot interpretation, where appropriate we have now highlighted only the non-significant differences and refer to the supplemental file for more detail of the precise statistical significance in all figure legends. The over-accurate numbers have also been changed to a classical * based system.*

5) The number of proteins in the bins in 4B is not the number of mRNAs in Fig. 4A. Could the authors state the size of the bins in Figure 4B either in the Table of Figure 4A or in 4B again.

*These numbers have now been added. The number for the mRNAs is greater than for the number of proteins as the RNA-seq is a more sensitive technique.*

6) u should be correct for µ

*This has been corrected.*

Requested comments:

The pSILAC experiment seems ok. Suppl. Table 4 should not just contain the gene names, but also the unique identifiers such as Uniprot or whatever was used for the MQ search. Especially as MQ output results in proteinGroups. Additionally, it would be helpful to have the proteinGroups intensity data in this table as well.

*The table has now been updated to contain this additional information.*

*Rebuttal references:*

*Bensaude O. Inhibiting eukaryotic transcription: Which compound to choose? How to evaluate its activity? Transcription. 2011;2(3):103–8.*

*Duncan CDS, Mata J. Effects of cycloheximide on the interpretation of ribosome profiling experiments in Schizosaccharomyces pombe Sci Rep. 2017;7(1):1–11. A*

*Hussmann JA, Patchett S, Johnson A, Sawyer S, Press WH. Understanding Biases in Ribosome Profiling Experiments Reveals Signatures of Translation Dynamics in Yeast. Snyder M, editor. PLOS Genet. 2015 Dec 11;11(12):e1005732.*

*Ingolia NT, Lareau LF, Weissman JS. Ribosome profiling of mouse embryonic stem cells reveals the complexity and dynamics of mammalian proteomes. Cell. 2011 Nov 11;147(4):789–802.*

*Ito K, Takahashi A, Morita M, Suzuki T, Yamamoto T. The role of the CNOT1 subunit of the CCR4-NOT complex in mRNA deadenylation and cell viability. Protein Cell. 2011 Sep;2(9):755–63.*

*Raisch T, Sandmeir F, Weichenrieder O, Valkov E, Izaurralde E. Structural and biochemical analysis of a NOT1 MIF4G-like domain of the CCR4-NOT complex. J Struct Biol. 2018 Dec 1;204(3):388–95.*

*Santos DA, Shi L, Tu BP, Weissman JS. Cycloheximide can distort measurements of mRNA levels and translation efficiency. Nucleic Acids Res. 2019 Jun 4;47(10):4974–85.*

*Sharma P, Nilges BS, Wu J, Leidel SA. The translational inhibitor cycloheximide affects ribosome profiling data in a species-specific manner bioRxiv 2019*

*Timmers HTM, Tora L. Transcript Buffering: A Balancing Act between mRNA Synthesis and mRNA Degradation. Mol Cell. 2018 Oct 4;72(1):10–7.*

**Second round of review**

**Reviewer 1**

The authors have gone to considerable effort to clarify and respond to the majority of my suggestions concerning additional experiments. As a result, the authors have improved the manuscript and clarified several questions.

Just one small detail:

Can the authors clarify if the changes in TE observed for the GC-ending codons rich R-Luc is a consequence of only changes in translation, or are there also changes in RNA levels (or both) upon CNOT1 depletion (luciferase activity and RNA levels graphs could be provided)?

I consider that the manuscript merits publication in Genome Biology.

**Reviewer 2**

The authors have addressed my comments satisfactorily. However, there are still a few "u" instead of "micro" in the manuscript (e.g. SuppFigure 1). I also would like to note that the codon bias issue is not resolved and the paper they cite is a preprint. However, I think the manuscript scientifically improved and can be accepted.

**Authors Response**

**Point-by-point responses to the reviewers’ comments:**

We have now completed all the editorial changes requested, added to the text in response to reviewer 1s final comment and we have ensured all data and code have been made publicly available and correctly referenced.
